# Engineered Au@CeO_2_ Hybrid Nanoparticles With Microenvironment Dependent Self‐Adjustability for Integration of Tumor‐Specific Photothermal‐Chemodynamic Therapy and Inflammation Prevention

**DOI:** 10.1002/advs.76172

**Published:** 2026-06-22

**Authors:** Wenyun Mu, Wenjuan Tang, Handan Zhang, Jie Liu, Jiaqi Zhang, Yu Yao, Xiao Fu, Xin Chen, Yanmin Zhang

**Affiliations:** ^1^ Department of Medical Oncology The First Affiliated Hospital of Xi'an Jiaotong University Xi'an Shaanxi P. R. China; ^2^ Department of Chemical Engineering Shaanxi Key Laboratory of Energy Chemical Process Intensification Institution of Polymer Science in Chemical Engineering School of Chemical Engineering and Technology Xi'an Jiaotong University Xi'an P. R. China; ^3^ Department of Gastroenterology The First Affiliated Hospital of Xi'an Jiaotong University Xi'an Shaanxi P. R. China; ^4^ School of Pharmacy Health Science Center Xi'an Jiaotong University Xi'an P. R. China

**Keywords:** esterase and Ca^2+^ co‐responsiveness, intelligent Au@CeO_2_ hybrid nanoparticle, microenvironment dependent self‐adjustability, on‐demand inflammation prevention, tumor‐specific photothermal‐chemodynamic therapy

## Abstract

Although nanoparticle‐based photothermal therapy (PTT) and chemodynamic therapy (CDT) hold great promise for tumor treatment, their clinical translation remains limited by off‐target tissue damage and therapy‐induced peritumoral inflammation. To address these challenges, we engineered microenvironment‐adaptive Au@CeO_2_ hybrid nanoparticles (ACEF) with self‐adjustable structural and catalytic behaviors for integrating tumor‐specific PTT/CDT with inflammation prevention. In tumor regions, FA‐mediated accumulation and intracellular esterase/Ca^2+^ activation promote ACEF aggregation, thereby enhancing near‐infrared (NIR)‐responsive photothermal conversion and reactive oxygen species (ROS)‐generating catalytic activity for localized tumor inhibition. In contrast, in surrounding normal tissues, dispersed ACEF predominantly exhibits ROS‐scavenging behavior through Ce‐based redox regulation, helping reduce excessive oxidative stress and inflammatory responses. This spatially adaptive behavior enables ACEF to exert therapeutic ROS/heat generation mainly in tumor‐associated environments while maintaining antioxidant protection under physiological conditions. In vitro and in vivo results demonstrated that ACEF effectively suppressed primary tumor growth and lung metastasis, reduced abnormal oxidative/inflammatory responses in non‐tumor tissues, and showed no obvious systemic toxicity under the tested conditions. This work provides a microenvironment‐dependent self‐adjustable strategy for integrating tumor‐specific photothermal‐chemodynamic therapy with inflammation prevention.

## Graphical abstract

An intelligent Au@CeO_2_ Hybrid Nanoparticles (ACEF) with microenvironment‐dependent self‐adjustability was engineered to not only perform efficient and tumor‐specific photothermal‐chemodynamic therapy for precise tumor elimination, but also serve as a ROS scavenger in healthy tissues around the tumor to avoid the unexpected oxidative damage and inflammation, which presents a promising strategy for the highly effective and safe treatment of malignant tumors.

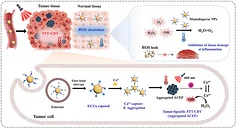

## 1 Introduction

The clinical management of malignant tumors continues to face formidable challenges despite decades of therapeutic advancements [1]. Conventional modalities, including surgery, chemotherapy, and radiotherapy, often inflict irreversible damage to healthy tissues, ultimately compromising patient survival and quality of life [2, 3, 4, 5]. To improve the therapeutic efficiency, novel strategies based on various nanomaterials such as chemodynamic therapy (CDT) [6, 7, 8] and photothermal therapy (PTT) [9, 10, 11] have been developed. Their catalytic function and thermal damage properties highly depend on the internal and external stimuli, such as H_2_O_2_ and NIR radiation, holding great promise for precise tumor treatment [12, 13, 14, 15, 16].

Although CDT, PTT, and their synergistic combination have opened new avenues to achieve tumor‐specific therapy with higher safety and efficiency, their specificity is compromised by the widespread presence of elevated H_2_O_2_ in inflammatory/ischemic tissues and the non‐specific distribution of photothermal agents in surrounding healthy tissues. This often leads to uncontrollable ROS generation and local heating beyond tumor regions to collateral damage to peritumoral tissues [17, 18, 19]. More critically, both of these therapies significantly exacerbate oxidative stress. CDT directly produces ROS that further diffuses into the surrounding tissues [20], while PTT triggers mitochondrial dysfunction and ROS imbalance, followed by ROS leakage [21, 22]. This dual‐source ROS overflow rapidly establishes an inflammatory microenvironment, which significantly promotes tumor metastasis [23, 24, 25]. Therefore, developing new nanomaterials with self‐adjustable functions to spatially confine the PTT and CDT therapeutic effects only within tumor lesions, while simultaneously serving as ROS scavengers in adjacent healthy tissue to prevent oxidative damage, would be a promising strategy for improving tumor management.

Cerium oxide (CeO_2_) nanoparticles may provide a potential solution to achieve the on‐demand manipulation of ROS through their unique redox duality [26]. The interconversion of Ce valence states between Ce^4+^ and Ce^3+^ enables disparate enzymatic behaviors of CeO_2_, which either acts as a peroxidase (POD) mimic to produce ROS, or serves as catalase (CAT) and superoxide dismutase (SOD) mimics to achieve ROS clearance [27, 28]. Therefore, spatially controlling the ratio of Ce^4+^ and Ce^3+^ in CeO_2_ nanoparticles would enable them to exert precise CDT in tumor cells and ROS clearance in surrounding healthy tissue, ultimately achieving on‐demand tumor damage without adverse inflammatory responses.

In our previous studies, various intelligent gold nanoparticles (Au NPs) were developed to achieve tumor‐specific PTT via stimuli‐responsive aggregation, taking advantage of the elevated levels of Ca^2+^, Cu^2+^, as well as overexpressed enzymes and miRNAs in tumor cells [29, 30, 31, 32], and the photothermal process has been demonstrated to drive the reduction of Ce^4+^ to Ce^3+^ via hot electron transfer [33]. Thus, the integration of Au NPs with CeO_2_ would be an effective approach to construct the ideal nanomaterial with controllable photothermal and catalytic functions, which could simultaneously perform tumor‐specific PTT and CDT only in tumor cells, along with inhibition of inflammation via ROS scavenging in the peritumoral microenvironment.

**SCHEME 1:**
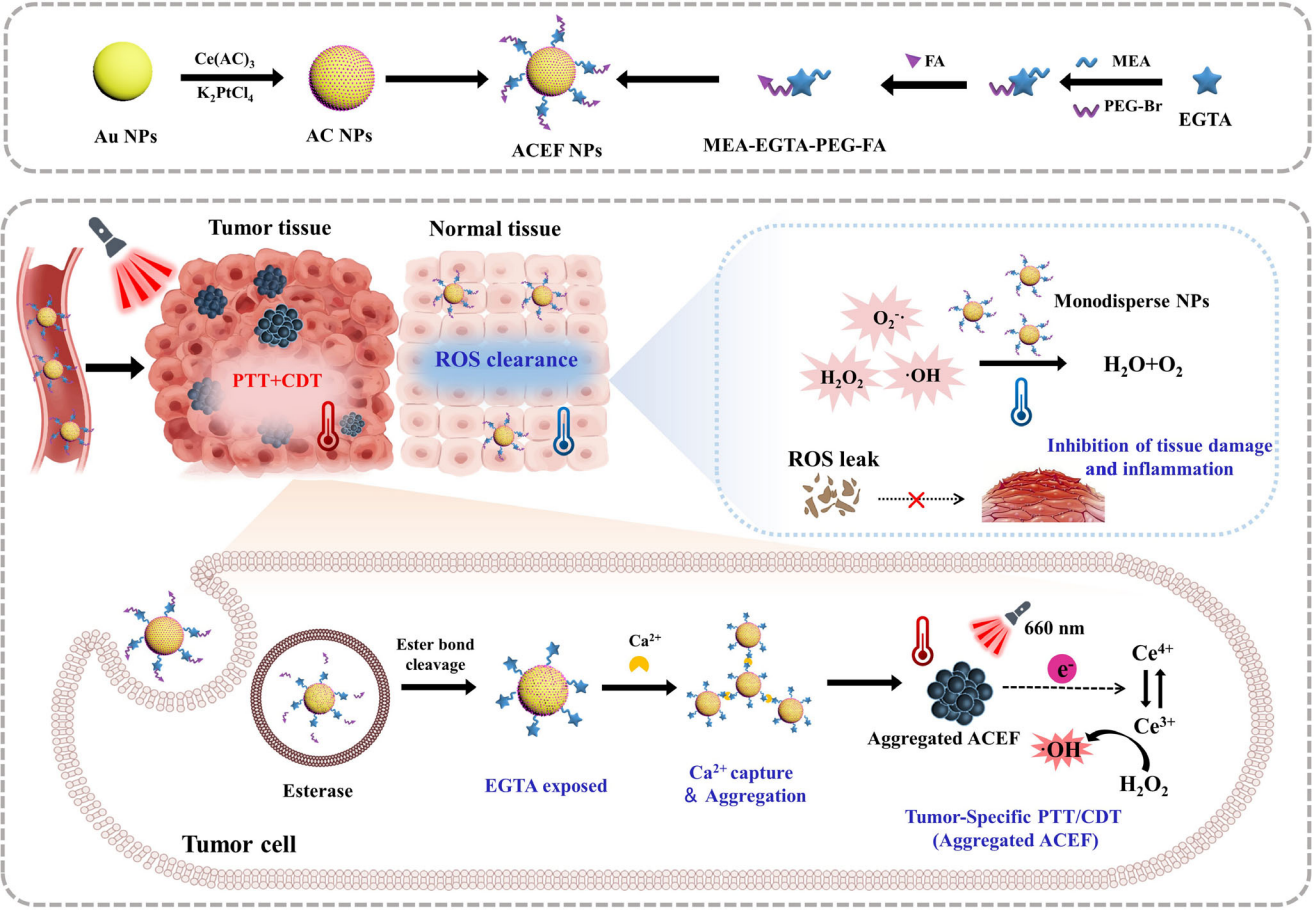
Schematic illustration of the synthesis and microenvironment‐adaptive therapeutic mechanism of Au@CeO_2_@MEA‐EGTA‐PEG‐FA (ACEF) hybrid nanoparticles. ACEF undergoes esterase/Ca^2+^‐associated aggregation in tumor‐associated environments to enhance near‐infrared (NIR)‐responsive photothermal‐chemodynamic therapy, while remaining mainly dispersed under physiological conditions to scavenge reactive oxygen species (ROS) and reduce excessive oxidative/inflammatory stress in adjacent normal tissues.

According to the above design concept, we fabricated an ethylene glycol bis(2‐aminoethyl ether)‐N,N,N’,N’‐tetraacetic acid (EGTA, a Ca^2+^ chelator [34]) and folic acid (FA, a tumor targeting agent [35, 36]) co‐modified Au@CeO_2_ hybrid nanoparticle (Au@CeO_2_@MEA‐EGTA‐PEG‐FA, ACEF for short), in which Ca^2+^ was selected as a trigger to induce the intratumoral PTT and CDT, considering the abnormally high Ca^2+^ levels in tumor tissues [37, 38]. To avoid the unexpected interference of systemic Ca^2+^, EGTA was covered by PEG via ester bonds to keep these nanoparticles inactive during blood circulation. After FA‐mediated tumor accumulation and endocytosis, the ester bonds on these nanoparticles would be rapidly cleaved by abundant intracellular esterases to expose EGTA [39, 40]. The released EGTA chelates intracellular Ca^2+^, inducing nanoparticle aggregation and enabling tumor‐specific PTT [29], while simultaneously down‐regulating Ca^2+^ to further suppress tumor progression by disrupting essential signaling pathways [41, 42, 43]. Meanwhile, the Au segments exhibit glucose oxidase (GOx)‐like activity to convert glucose into gluconic acid and H_2_O_2_ [44, 45]_,_ which initiates the POD‐like enzyme activity of CeO_2_ to generate ·OH for tumor‐specific CDT, along with the conversion from Ce^3+^ to Ce^4+^. More importantly, the PTT process efficiently generates hot electrons within ACEF aggregates to sustainably reduce Ce^4+^ back to Ce^3+^, which not only significantly enhances the therapeutic efficiency [46, 47, 48] but also provides further controllability for more precise therapy. In contrast, the Ce^4+^‐rich ACEF remains in a monodispersed state in the surrounding healthy tissues, which scavenges ROS caused by the CDT and PTT to inhibit the unexpected inflammation via CAT‐like and SOD‐like enzyme activities, regardless of the NIR irradiation [49, 50] (Scheme 1).

## 2 Results and Discussion

### 2.1 Preparation and Characterization of ACEF

The ACEF nanoparticles (NPs) were synthesized through a multi‐step process. Initially, Au NPs with an average diameter of approximately 15 nm were prepared via a sodium citrate reduction method [51]. Subsequently, a thin and highly dispersed CeO_2_ surface component was formed on Au NPs through the hydrolytic reaction of cerium acetate. To construct the esterase‐responsive Ca^2+^ ‐capture unit, mercaptoethylamine (MEA), ethylene glycol bis (2‐aminoethyl ether)‐N,N,N′,N′‐tetraacetic acid (EGTA), PEG‐Br, and folic acid (FA) were sequentially conjugated through classical amination and esterification reactions, resulting in the formation of a terminal sulfhydryl‐containing complex (MEA‐EGTA‐PEG‐FA). This complex was then grafted onto the surface of the Au@CeO_2_ (AC) NPs via Au‐S bonding to yield the final ACEF NPs. The chemical structure of MEA‐EGTA‐PEG‐FA was confirmed by ^1^H nuclear magnetic resonance (^1^H NMR) spectroscopy (Figure S1), which exhibited the characteristic signals corresponding to PEG, MEA, EGTA, and FA, thereby verifying the successful synthesis of the MEA‐EGTA‐PEG‐FA.

**FIGURE 1:**
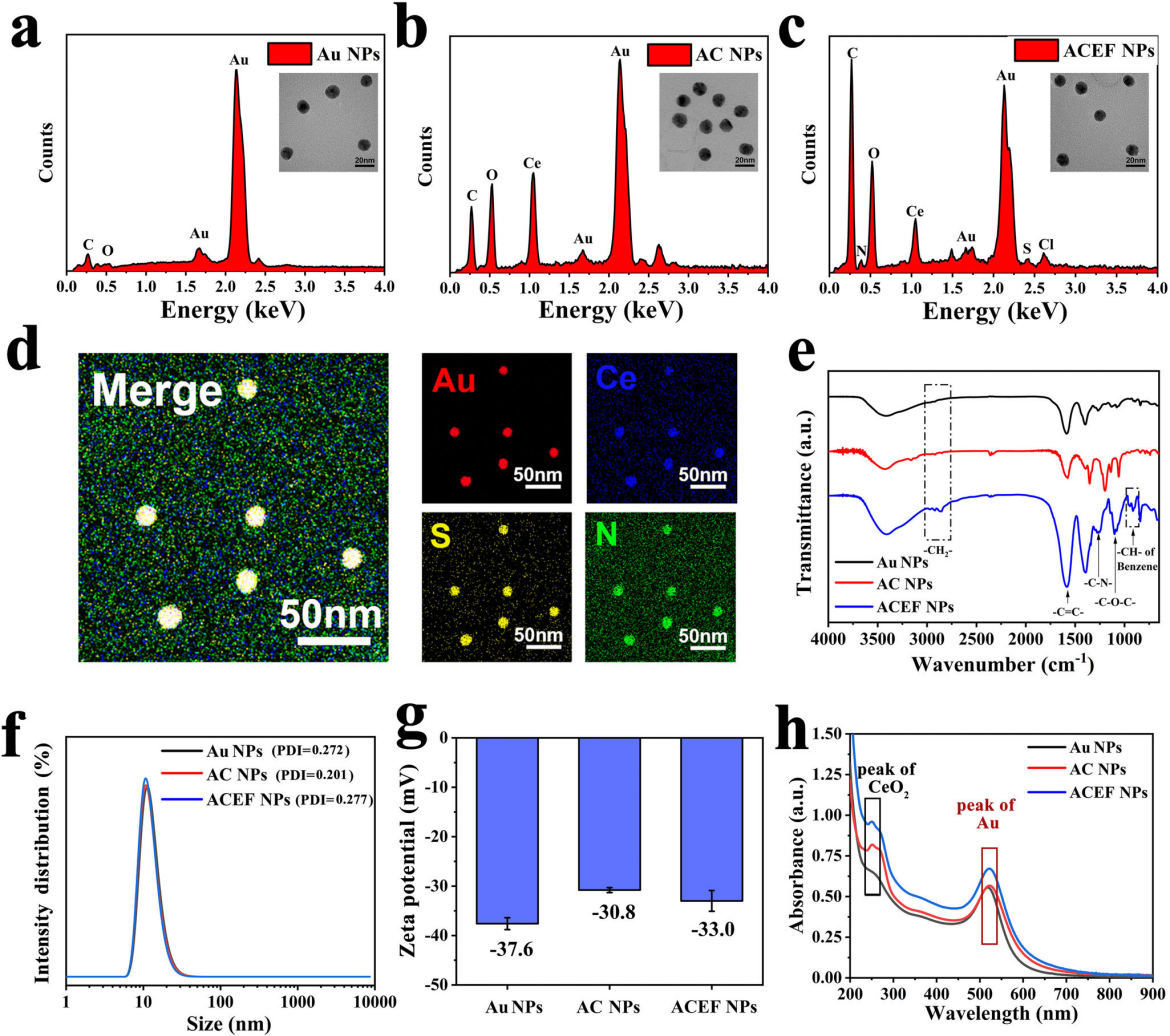
(a–c) Transmission electron microscope (TEM) images and energy dispersive X‐ ray spectroscopy (EDS) of Au NPs, AC NPs, and ACEF NPs. (d) Element mapping images of ACEF NPs. (e) Fourier‐ transform infrared (FTIR) spectra of the Au NPs, AC NPs, and ACEF NPs. (f) Dynamic light scattering (DLS), (g) Zeta potential results, and (h) UV–vis spectrometry of the Au NPs, AC NPs_,_ and ACEF NPs. Error bars represent mean ± SD (n = 3).

The morphology and chemical composition of the synthesized ACEF NPs and their intermediates were systematically characterized using transmission electron microscopy (TEM) and energy‐dispersive X‐ray spectroscopy (EDS). As shown in Figure 1a–c, Au NPs, AC NPs, and ACEF NPs all exhibited a spherical structure with an average diameter of approximately 15 nm. It was worth noting that the characteristic elements, including Ce (from CeO_2_), N (from MEA‐EGTA‐PEG‐FA), and S (from MEA‐EGTA‐PEG‐FA), were only detected in the gradually synthesized nanoparticles as expected. Notably, the size of AC NPs remained essentially unchanged compared to the pristine Au NPs, suggesting that the CeO_2_ modification did not form a thick, distinct shell, but rather a very thin or highly dispersed layer on the Au surface. The EDS mapping results (Figure 1d) further showed that the Ce signal spatially overlapped with the Au nanoparticle region rather than being randomly distributed in the background, indicating that the Ce‐containing component was associated with Au NPs instead of existing as separated CeO_2_ particles. To further examine the local structure of the AC hybrid nanoparticles, HRTEM and FFT analyses were performed (Figure S2). Clear lattice fringes with an interplanar spacing of approximately 0.235 nm were observed in the nanoparticle core, corresponding to the Au (111) plane. The FFT pattern of the core region also showed distinct diffraction spots, confirming the crystalline nature of the Au core. In contrast, the peripheral region around the Au core exhibited a low‐contrast layer with unclear and discontinuous lattice features, and the corresponding FFT pattern displayed diffuse features rather than sharp crystalline diffraction spots. These HRTEM/FFT characteristics suggest that the peripheral CeO_2_ component mainly exists as an amorphous or poorly crystalline layer rather than forming a thick and well‐crystallized CeO_2_ shell. X‐ray diffraction (XRD) analysis was further performed to characterize the crystallinity and phase composition of AC NPs (Figure S3). The diffraction pattern of AC NPs showed characteristic peaks of Au (JCPDS No. 04–0784) at 38.1°, 44.3°, 64.5°, 77.5°, and 81.7°, corresponding to the (111), (200), (220), (311), and (222) planes. However, no distinct diffraction peaks for CeO_2_ were observed, only a very weak and broad hump around 31°, which might be indicative of amorphous or poorly crystalline CeO_2_. To further evaluate the defect‐related electronic structure of the Au@CeO_2_ hybrid core, electron paramagnetic resonance (EPR) analysis was performed. As shown in Figure S4, AC exhibited a distinct resonance signal at g = 2.0014, which falls within the commonly reported range of oxygen‐vacancy‐related EPR signals in CeO_2_‐based materials. This signal was assigned to unpaired electrons trapped in oxygen vacancies or defect‐related electronic states, supporting the existence of oxygen‐vacancy or defect‐related sites in the CeO_2_ component of AC. Inductively coupled plasma‐mass spectrometry (ICP‐MS) quantification provided a quantitative support for these observations. The results revealed that the mass percentage of Ce in AC NPs was 1.65 ± 0.14 wt.%. However, the characterization results suggest that CeO_2_ was mainly present as an ultrathin and highly dispersed surface phase on Au nanoparticles rather than as a bulk ceria domain. Since the relevant redox reactions mainly occur at exposed surface Ce sites and Au‐CeO_2_ interfacial regions, this type of distribution can still provide accessible active sites despite the low total Ce content. In addition, such a thin CeO_2_ layer helps preserve the plasmonic property of Au for the subsequent LSPR coupling and NIR‐associated catalytic enhancement. Combined with the unchanged particle size observed in TEM, these results suggest that the CeO_2_ component is present in a very small amount and likely exists as a thin amorphous layer or highly dispersed clusters on the Au surface, rather than forming a significant crystalline shell.

The stepwise synthesis of ACEF NPs was further investigated using Fourier‐transform infrared (FTIR) spectroscopy (Figure 1e). The characteristic vibrations of ACEF NPs corresponding to ‐CH_2_‐, ‐C═C‐, ‐C‐N, ‐C‐O‐C‐, and the benzene ring skeleton confirmed the successful modification with MEA‐EGTA‐PEG‐FA. To further clarify the composition of ACEF NPs, TGA was used to estimate the total amount of grafted organic components. As shown in Figure S5, AC showed only slight weight loss during heating, whereas ACEF exhibited an obvious additional weight loss mainly at approximately 280–400°C, corresponding to the decomposition of the grafted MEA‐EGTA‐PEG‐FA organic layer. Based on the residual mass difference between AC and ACEF at high temperature, the total organic ligand loading in ACEF was estimated to be 16.47 wt.%. According to the theoretical molecular composition of MEA‐EGTA‐PEG‐FA, the EGTA and FA contents in ACEF were further estimated to be approximately 1.06 wt.% and 1.23 wt.%, respectively. These results further support the successful surface modification of AC with the EGTA/FA‐containing organic layer. To assess the colloidal stability and hydrodynamic behavior, the dynamic light scattering (DLS) measurement was carried out. As shown in Figure 1f, these nanoparticles (Au NPs, AC NPs, and ACEF NPs) maintained a consistent particle size (∼15 nm), aligning closely with TEM observations, which confirmed that these nanoparticles could maintain monodispersity during the stepwise modification processes.

Meanwhile, ACEF NPs showed a negative surface charge (−33.0 mV) (Figure 1g), which was crucial for minimizing nonspecific protein adsorption, such as serum albumin, during systemic circulation, as further supported by the bovine serum albumin (BSA) binding assays (Figure S6). Specifically, ACEF NPs exhibited negligible interaction with BSA and did not show a clear tendency to bind to BSA after co‐incubation, suggesting the high stability of ACEF NPs in physiological environments. Finally, UV–vis spectroscopy (Figure 1h) provided key insights into the optical properties of ACEF NPs. The distinct absorption peak observed at 249 nm confirmed the successful integration of CeO_2_, while the absence of absorption in the long wavelength (> 660 nm) region indicated that the ACEF NPs are in a photothermal‐off state under physiological conditions. This design could ensure that photothermal activity is mainly activated by Ca^2+^‐induced aggregation in tumor‐associated environments, thereby avoiding unintended thermal damage during the blood circulation process. Taken together, all these results demonstrated that ACEF NPs constitute a precisely engineered platform with tailored structural, colloidal, and optical properties for tumor‐specific therapy.

### 2.2 Esterase‐Responsive Ca^2+^ Capture and Ca^2+^ Dependent Photothermal Effects of ACEF

**FIGURE 2:**
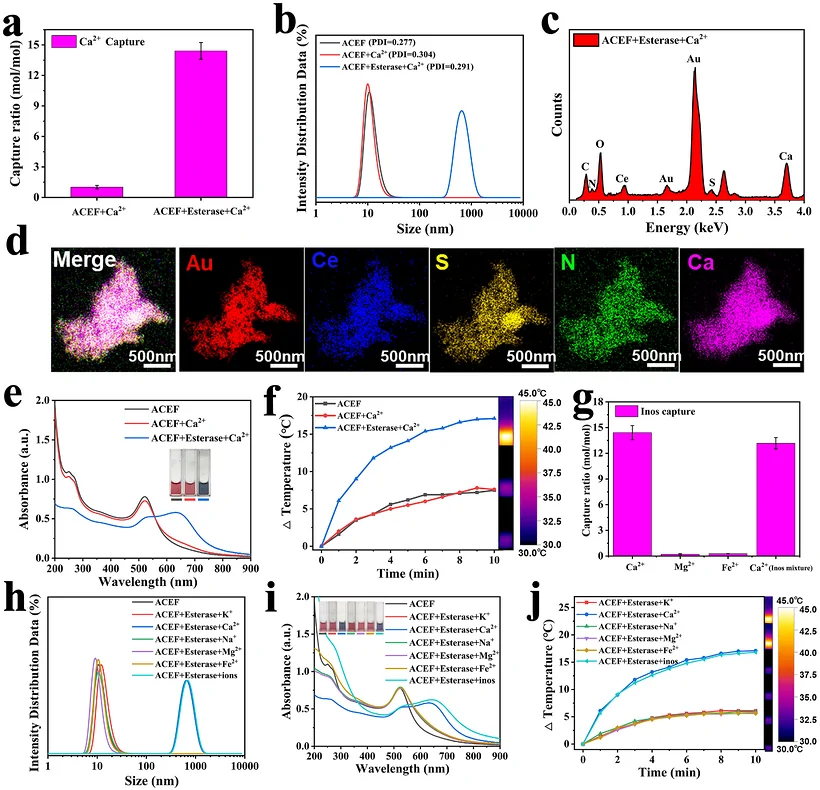
(a) Esterase‐dependent Ca^2+^ capture and (b) Ca^2+^ capture‐induced aggregation of ACEF NPs measured in PBS containing Ca^2+^ with and without esterase. (c) Energy dispersive X‐Ray spectroscopy (EDS) and (d) Element mapping images of ACEF aggregates after the esterase‐dependent Ca^2+^ capture measured in PBS containing Ca^2+^ and esterase. (e) UV–vis spectrometry and photos, as well as (f) Photothermal curves and corresponding photothermal image of ACEF NPs in PBS containing Ca^2+^ with and without esterase. (g) Specific Ca^2+^ capture of ACEF NPs was measured in the solution containing either a single ion (Ca^2+^, Fe^2+,^ and Mg^2+^) or the ion mixture with both Ca^2+^, Fe^2+,^ and Mg^2+^. (h) Dynamic light scattering (DLS), (i) UV–vis spectrometry and photos, (j) Photothermal curves and the corresponding photothermal image of ACEF NPs in PBS with different ions (Ca^2+^, Fe^2+^, Mg^2+^, Na^+^, K^+^) or their mixture. Error bars represent mean ± SD (n = 3).

The function of EGTA can be temporarily masked in normal cells due to the ester bond between EGTA and PEG within the MEA‐EGTA‐PEG‐FA functional molecule. Overexpression of esterases in tumor cells would cleave the ester bond between EGTA and PEG, exposing the carboxyl groups of EGTA and thereby activating the Ca^2+^‐chelating ability of EGTA. To validate the tumor microenvironment (TME)‐responsive functionality of ACEF nanoparticles, we first investigated their Ca^2+^ capture capability. As shown in Figure 2a, atomic absorption spectroscopy (AAS) revealed that 1 mol of ACEF captured 14.41 mol of Ca^2+^ in the presence of esterase. In contrast, negligible Ca^2+^ capture was observed in the absence of esterase, unequivocally confirming that the Ca^2+^ capture function of EGTA was esterase‐dependent. To further explore the consequences of Ca^2+^ capture, DLS measurement was carried out (Figure 2b). Compared with simulated environments without Ca^2+^ or esterase, a significant increase in the hydrodynamic diameter of ACEF was observed exclusively under the simulated TME conditions (esterase + Ca^2+^). This aggregation behavior was further corroborated by EDS (Figure 2c), where a distinct Ca peak emerged in the spectrum of ACEF after exposure to esterase and Ca^2+^, verifying the incorporation of Ca^2+^. This morphological transformation could be better visualized using transmission electron microscopy (Figure 2d). The Ca^2+^‐triggered aggregation of ACEF NPs was strategically designed to activate PTT functionality. UV–vis spectroscopy (Figure 2e) revealed a redshift in the localized surface plasmon resonance (LSPR) peak of Au NPs from 520 nm (monodispersed state) to 660 nm (aggregated state), specifically in the presence of esterase and Ca^2+^. This spectral shift translated into enhanced photothermal conversion under NIR irradiation, as evidenced by the rapid temperature increase (ΔT > 15°C) observed in the ACEF + esterase + Ca^2+^ solutions group (Figure 2f). In contrast, control groups lacking either esterase or Ca^2+^ showed negligible temperature changes, highlighting the precision of this TME‐activated PTT strategy.

To further evaluate the selectivity of ACEF in complex physiological environments, we examined its behavior in the presence of different divalent ions. As shown in Figure 2g, AAS results demonstrated that ACEF preferentially captured Ca^2+^ even in solutions containing Ca^2+^, Mg^2+^, and Fe^2+^, whereas the capture of other ions remained minimal. Consistently, DLS (Figure 2h) and UV–vis spectroscopy (Figure 2i) showed that only Ca^2+^‐containing solutions induced obvious aggregation and LSPR redshift. Corresponding photothermal measurements (Figure 2j) further showed that selective temperature elevation occurred only in these groups. To quantitatively compare the photothermal properties of different materials, photothermal conversion efficiency (PCE) was measured for AC, ACEF, and ACEF exposed to different ions. As shown in Figure S7, AC exhibited a PCE of approximately 10%, whereas surface‐functionalized ACEF showed a relatively lower PCE of 6.5%, likely because the surface modifiers partially shielded the light‐absorbing core. Notably, ACEF treated with esterase and Ca^2+^ showed significantly enhanced PCE, whereas other ions caused no detectable change. These results further support that Ca^2+^‐triggered aggregation is the major factor responsible for enhancing the photothermal performance of ACEF.

### 2.3 On‐Demand ROS Manipulation of ACEF Based on Its Adjustable Enzymatic Activities

Building upon the tumor‐selective Ca^2+^ capture and NIR‐enhanced photothermal conversion demonstrated in Figure 2, we further investigated the oxidoreductase properties of ACEF, as well as its coordination with TME‐specific ROS generation and ROS‐scavenging capacity for protecting normal tissue.

**FIGURE 3:**
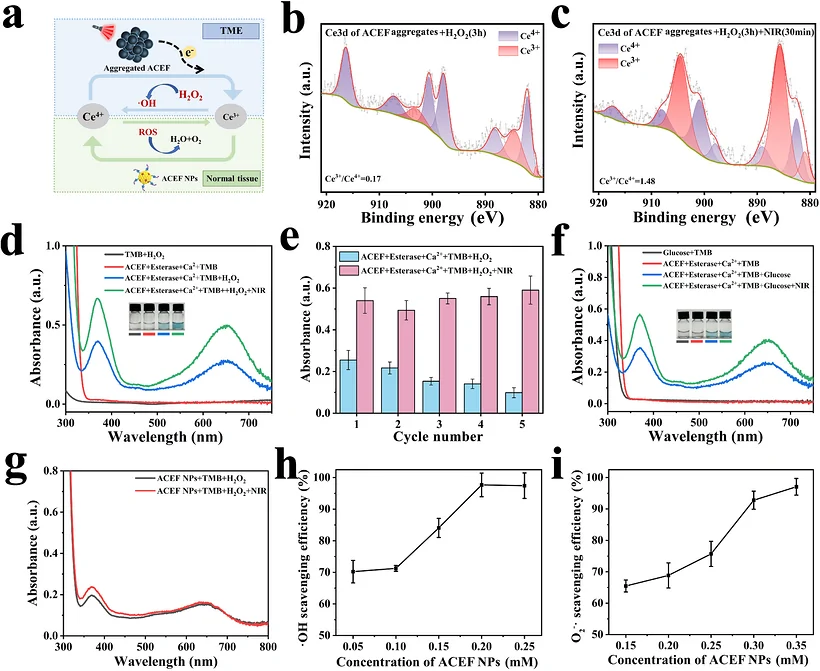
(a) Schematic of tumor‐specific microenvironment (Over‐expressed Ca^2+^ and esterase) dependent catalytic duality: ACEF aggregates under NIR irradiation promote Ce^4+^ to Ce^3+^ regeneration to enhance ·OH production via peroxidase (POD) activities, while dispersed ACEF NPs with plenty of Ce^4+^ in surrounding normal tissues scavenge ROS via catalase (CAT) and superoxide dismutase (SOD) activities. (b‐c) XPS spectra and quantified Ce^3+^/Ce^4+^ ratios of ACEF aggregates (with H_2_O_2_) without and with NIR irradiation. (d) ROS generation of ACEF under different conditions (PBS with and without H_2_O_2_ + NIR radiation) was measured by UV spectra and photographs using TMB as ROS indicator. (e) Catalytic stability of ACEF aggregates and ACEF aggregates +NIR over five cycles, indicating the sustained conversion from Ce^4+^to Ce^3+^. (f) ROS generation of ACEF NPs in PBS with or without glucose and NIR irradiation was measured by UV spectra and photographs using TMB as ROS indicator. (g) UV spectra and photographs of the monodispersed ACEF in PBS (pH = 7.4) containing H_2_O_2_ and TMB with and without NIR exposure, indicating the weak catalytic function of monodispersed ACEF even under NIR radiation. (h) The concentration‐dependent ·OH scavenging of monodispersed ACEF NPs at PBS (pH = 7.4). (i) The concentration‐dependent O_2_^−^· scavenging of monodispersed ACEF NPs at PBS (pH = 7.4). Error bars represent mean ± SD (n = 3).

ACEF exhibited a pronounced pH‐dependent ROS‐generating behavior, with markedly higher ROS production under acidic conditions than under neutral conditions (Figure S8), indicating that acidic microenvironments are more favorable for activating its POD‐like catalytic function. In acidic TME, ACEF aggregates exhibit POD‐like enzymatic activity, where CeO_2_ catalyzes the decomposition of H_2_O_2_ into hydroxyl radicals (·OH) through Ce^3+^‐mediated Fenton‐like reactions. Critically, NIR irradiation induces hot electron generation during photothermal conversion, which drives the reduction of Ce^4+^ to Ce^3+^, thereby regenerating catalytically active sites and amplifying ·OH production (Figure 3a). To further confirm the mechanism underlying the advantage of the Au@CeO_2_ hybrid nanoparticle structure, we performed a series of in vitro TMB‐based colorimetric assays to systematically evaluate the POD‐like activity and ROS generation efficiency of different formulations (Figure S9). First, we directly compared the ROS production capacity of the aggregated AC hybrid nanoparticles and the aggregated physical Au+CeO_2_ mixture under standard conditions without NIR irradiation (Figure S9a). This result suggests that the integrated hybrid nanoparticle structure enables stronger electronic interaction between Au and CeO_2_ components, thereby synergistically enhancing the POD‐like activity of CeO_2_, whereas such a cooperative effect is absent in the physically mixed system where Au and CeO_2_ exist as independent phases. To further explore the effect of NIR irradiation on ROS generation, we separately assessed the performance of the aggregated physical Au+CeO_2_ mixture (Figure S9b) and the aggregated AC hybrid nanoparticles (Figure S9c) with and without NIR irradiation. The data demonstrated that the free mixture only exhibited a marginal increase in ROS levels upon NIR irradiation, while the AC hybrid nanoparticles showed a significant NIR‐dependent enhancement of ROS production, further confirming that both the acidic microenvironment and the integrated hybrid nanoparticle structure are critical for efficient ROS amplification.

To validate the mechanism of Ce valence state cycle‐mediated CDT, X‐ray photoelectron spectroscopy (XPS) was employed to track the valence transition of Ce. As shown in Figure 3b,c, the Ce^3+^/Ce^4+^ ratio in ACEF aggregates (co‐incubated with H_2_O_2_) was 0.17 in the absence of NIR irradiation and increased to 1.43 upon NIR irradiation. This valence transition directly enhanced the POD‐like activity, as evidenced by TMB chromogenic assays: even without NIR irradiation, ACEF aggregates generated moderate ·OH (likely due to basal Ce^3+^‐mediated catalytic reaction), and continuous NIR irradiation doubled ·OH production (Figure 3d). To further clarify the quantitative correlation between the Ce^3+^/Ce^4+^ valence state cycle and CDT efficiency, we supplemented dynamic tracking experiments (Figure S10). Time‐dependent XPS analysis (Figure S10a) showed that the Ce^3+^/Ce^4+^ ratio of ACEF gradually decreased from 1.43 to 0.17 with the prolonged co‐incubation time with H_2_O_2_ (30 min, 1 h, 3 h), indicating continuous oxidation of Ce^3+^ to Ce^4+^ during the Fenton‐like reaction. After subjecting to NIR laser irradiation for different durations (5, 10, and 30 min) post‐co‐incubation, the Ce^3+^/Ce^4+^ ratio recovered to 0.36, 0.95, and 1.48, respectively, confirming that photothermal treatment can effectively regenerate Ce^3+^ and reactivate the Ce valence cycle. Correspondingly, time‐dependent ·OH detection via TMB colorimetric assay (Figure S10b) (post‐irradiation detection, distinct from the continuous irradiation ROS assay in Figure 3d) revealed that the ·OH generation efficiency was positively correlated with the Ce^3+^/Ce^4+^ ratio: it decreased with Ce^3+^ oxidation during ACEF‐H_2_O_2_ co‐incubation and increased with Ce^3+^ regeneration after photothermal treatment. To further determine whether this Ce valence‐state change was mainly caused by NIR irradiation rather than acidic pH or GSH in the tumor microenvironment, additional Ce 3d XPS analysis was performed using H_2_O_2_‐treated ACEF aggregates incubated for 30 min under tumor‐relevant acidic pH and GSH‐containing conditions, followed by Ce^3+^/Ce^4+^ ratio quantification. As shown in Figure S11, the tumor‐relevant pH/GSH treatment only slightly changed the Ce^3+^/Ce^4+^ ratio from 0.17 to 0.19, indicating that acidic pH and GSH alone did not induce obvious Ce^3+^ regeneration under the tested conditions. Together, these results demonstrated that the Ce^3+^/Ce^4+^ valence cycle acted as the core driving force for ACEF‐mediated CDT, and photothermal treatment can sustain and enhance the continuous CDT efficiency by facilitating Ce^3+^ regeneration. Crucially, ACEF+NIR maintained >90% catalytic efficiency over five cycles (Figure 3e), demonstrating self‐sustaining enzymatic catalytic loops enabled by NIR‐triggered Ce^3+^ regeneration. In contrast, the catalytic activity of ACEF without NIR irradiation gradually decreases over repeated cycles, suggesting that the regeneration of Ce^3+^ is limited in the absence of photothermal stimulation.

Remarkably, ACEF may partially overcome the intrinsic limitations of the tumor microenvironment through adaptive ROS amplification. In a glucose‐rich solution that simulates tumor metabolism, the addition of ACEF decreased the solution pH from 6.8 to 5.1 (Figure S12), and the reduced pH could further enhance the POD‐like enzymatic activity of CeO_2_. In addition, TMB oxidation was observed even in the absence of exogenous H_2_O_2_ (Figure 3f), suggesting that the Au component may contribute to in situ H_2_O_2_ generation through glucose oxidation. This process may further support ROS production in the simulated tumor environment. To further evaluate whether the NIR‐associated catalytic enhancement of ACEF could be retained under biologically relevant competitive conditions, we evaluated ROS generation with and without NIR irradiation in the presence of increasing GSH concentrations (Figure S13a), as well as under normoxic, hypoxic, and nearly anoxic conditions (Figure S13b). In addition, we further tested the combined extreme condition of high GSH (10 mM) and low dissolved oxygen (0.5 mg/L, Figure S13c). These results obviously showed significant NIR‐induced enhancement of catalytic efficiency in all test groups, even under the most stringent reductive and hypoxic environment, which provides direct evidence to support the therapeutic mechanism of ACEF NPs.

In contrast to the ROS‐generating mode of ACEF aggregates in acidic TME, dispersed ACEF NPs not only showed negligible ROS generation activity in simulated normal tissue (pH = 7.4) to avoid ROS damage to surrounding healthy tissue (Figure 3g), but served as a ROS‐scavenging agent to effectively neutralize multiple oxidative species through enzyme‐mimetic cascades. As shown in Figure 3h, the scavenging of ·OH increased with the rise of ACEF NPs concentration, and a 95% scavenging rate could be achieved at a concentration of only 0.2 mM, which was quantified using the salicylic acid trapping method. For O_2_^−^· elimination, nitro blue tetrazolium (NBT) photoreduction assays revealed that the O_2_^−^· scavenging efficiency of ACEF NPs is also proportional to its concentration, indicating their ability to effectively remove the O_2_^−^· as expected (Figure 3i). To in situ monitor the dynamic clearance of ^1^O_2_ by ACEF NPs, the photosensitizer Ce6 was used to generate ROS under 660 nm radiation, and 1,3‐diphenylisobenzofuran (DPBF) was monitored by recording the decrease in absorbance at 410 nm. As can be seen from Figure S14a, Ce6 continuously generated ^1^O_2_ under irradiation, which rapidly oxidized DPBF, causing a characteristic absorbance decline at 410 nm against time. As expected, the ACEF NPs addition significantly slowed the absorbance decay rate, clearly indicating its ability to consume ^1^O_2_ (Figure S14b). This could be attributed to the SOD‐mimetic property and the CAT‐mimetic property of ACEF NPs. Initially, the CeO_2_ component in ACEF acts as a SOD‐mimetic enzyme to convert O_2_^−^· to H_2_O_2_, then it further serves as a catalase‐like catalyst to decompose H_2_O_2_ into H_2_O and O_2_, thereby preventing oxidative chain reactions. All these results indicated that our ACEF is able to perform heterogeneous enzyme activities under different conditions. Its aggregated state in TME would couple photothermal‐enhanced valence cycling with self‐supplied H_2_O_2_ to generate a ROS storm for tumor‐specific therapy, while its dispersed state in normal tissues would maintain redox homeostasis via Ce^3+^/Ce^4+^ interconversion for inflammation prevention.

As an intravenous material used for tumor therapy, the stability of ACEF in organisms is also important. The structural and functional stability of ACEF under physiological conditions (PBS, pH 7.4) was comprehensively validated to ensure its application potential as an intravenous therapeutic agent. To further evaluate long‐term stability under physiological environments that mimic in vivo conditions (including serum proteins), we performed stability studies of ACEF in PBS (pH 7.4) and 5% FBS (simulating serum protein environment) at 37°C for 24, 48, and 72 h, respectively. Its physicochemical stability was characterized via DLS, TEM, and zeta potential measurements. Structural stability assessments via UV–vis spectroscopy (Figure S15) confirmed that ACEF maintained consistent optical properties over 72 h, demonstrating resistance to aggregation and degradation in blood‐like physiological environments. DLS results (Figure S16a) showed the average hydrodynamic diameter of ACEF remained stable at approximately 15 nm within 72 h of incubation in both PBS and 5% FBS, with no significant aggregation or size increase observed. The zeta potential of fresh ACEF was approximately −33 mV (Figure S16b). TEM characterization (Figure S16c) further confirmed that ACEF maintained its initial core‐shell nanostructure and monodisperse state after 72 h of incubation in both media, and no obvious morphological changes were observed compared with the fresh sample. After incubation in 5% FBS‐containing PBS (pH 7.4) solution at 37°C for 24, 48, and 72 h, the zeta potential only exhibited slight negative to fewer negative shifts. These minor changes in zeta potential were attributed to the non‐specific adsorption of a small amount of serum proteins on the nanoparticle surface, but no drastic fluctuations occurred, and the potential difference was small, indicating that the surface properties of ACEF remained stable and no significant structural damage induced by protein corona was observed. Functional analyses further revealed that ACEF retained favorable tumor‐targeting capabilities after 72 h incubation: ACEF preserved esterase‐responsive Ca^2+^ capture selectivity (Figure S17), Ca^2+^‐induced aggregation behavior (Figure S18), and NIR‐triggered photothermal conversion efficiency (Figure S19), while avoiding nonspecific binding with physiological divalent ions (Figure S20). Concurrently, ROS regulatory functions remained intact: NIR irradiation induced POD‐like activity for ·OH generation (Figure S21), while under neutral physiological conditions, the Ce^3+^/Ce^4+^‐mediated ROS scavenging was activated, effectively eliminating ·OH (Figure S22) and O_2_^−^· (Figure S23) at rates comparable to freshly prepared ACEF. To further distinguish the designed esterase/Ca^2+^‐triggered aggregation from nonspecific colloidal aggregation, ACEF was further incubated for 7 days under different non‐triggering conditions, including pH 7.4, pH 6.8, FBS‐containing medium, and high ionic strength solution containing 300 mM NaCl. As shown in Figure S24, DLS and zeta‐potential results showed no obvious hydrodynamic size increase or surface charge collapse during the 7‐day incubation. Consistently, UV–vis spectra showed no obvious long‐wavelength redshift or plasmon band broadening under these conditions, indicating that ACEF remained mainly monodispersed. These results suggest that ACEF aggregation is not caused by nonspecific pH variation, serum protein adsorption, or ionic strength effects, but depends on the designed esterase/Ca^2+^‐associated activation process. These collective findings underscored ACEF's robustness in physiological conditions, enabling sustained tumor‐specific Ca^2+^ trapping, photothermal activation, and ROS regulation without off‐target functional decay, thereby supporting its potential for prolonged therapeutic efficacy in vivo.

### 2.4 In Vitro Antitumor Activities of ACEF

**FIGURE 4:**
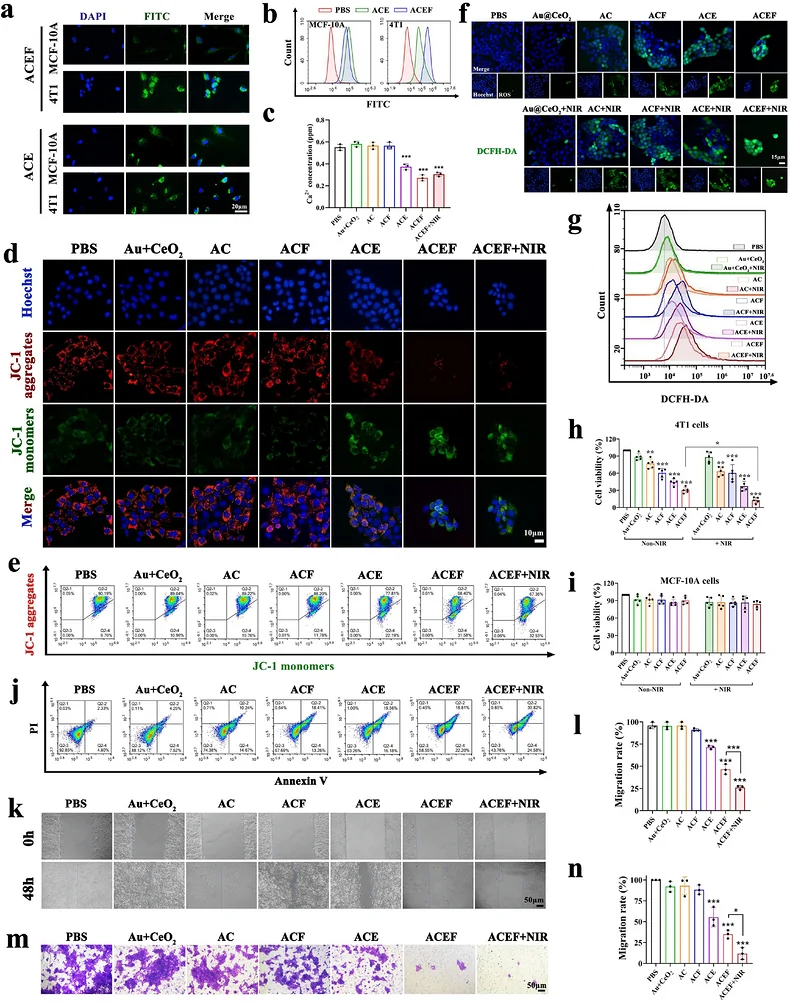
(a) Representative fluorescent images and (b) Flow cytometry analysis of breast cancer 4T1 cells and normal mammary epithelial MCF‐10A cells after incubation with PBS, ACE, and ACEF for 8 h. The scale bar is 20 µm. (c) Ca^2+^ concentration in the supernatant of 4T1 cells from different treatment groups. (d) Representative fluorescent images (The scale bar is 10 µm.) and (e) Flow cytometry analysis of JC‐1 probe in 4T1 cells under different treatment conditions. (f) Representative fluorescence images and (g) Flow cytometry analysis of 4T1 cells after treatment with different nanoparticles stained with DCFH‐DA for measuring intracellular ROS levels. The scale bar is 15 µm. (h, i) Cell viability of 4T1 cells (h) and MCF‐10A cells (i) after incubation with various groups (n = 5). (j) Flow cytometry analysis of 4T1 cells stained with Annexin V‐FITC/PI after treatment with different nanoparticles. (k,l) Wound healing assay and (m, n) transwell migration assay were conducted to evaluate the effects of the nanoparticles on the migratory capacity of 4T1 cells (n = 3). The scale bar is 50 µm. Error bars represent mean ± SD. ^(***^ *p* < 0.001, ^**^ *p* < 0.01, ^*^ *p* < 0.05).

To accurately assess the anti‐tumor activity of ACEF against malignant cancers, the specific uptake of these nanoparticles by tumor and normal cells was first investigated by fluorescence microscopy and flow cytometry after co‐incubation of ACEF with 4T1 cells (breast cancer cells) and MCF‐10A cells (non‐tumorigenic mammary epithelial cell line) for 8 h. The ACE group (Au@CeO_2_‐EGTA‐PEG) without folic acid (FA) conjugation served as a control group. As shown in Figure 4a, the green fluorescence intensity (FITC signal from ACEF) in 4T1 cells was much stronger than that in MCF‐10A cells after ACEF treatment, indicating significantly greater uptake by tumor cells. In contrast, both 4T1 cells and MCF‐10A cells exhibited similarly weak green fluorescence after co‐incubation with ACE (without FA). Quantitative analysis obtained by flow cytometry was displayed in Figure 4b and Figure S25, which confirmed these observations. Together, these results demonstrated that the excellent tumor selectivity of ACEF NPs was closely related to the effective recognition of over‐expressed folate receptors on tumor cell membranes [52, 53].

To further clarify the subcellular trafficking of ACEF following tumor cell internalization and its subsequent relationship with Ca^2+^ regulation, fluorescence colocalization assays were performed in 4T1 cells. ACEF was labeled with FITC, and cells were co‐stained with Hoechst (nuclear marker), LysoTracker Red (lysosomal marker), or MitoTracker Red (mitochondrial marker) for confocal laser scanning microscopy (CLSM) observations (Figure S26a). Quantitative analysis of the Pearson correlation coefficient (Figure S26b) revealed a time‐dependent subcellular distribution pattern of ACEF, which is consistent with our design rationale that ACEF does not directly target mitochondria but exerts its function through lysosome‐associated intracellular processing. At 45 min post‐incubation, ACEF‐FITC exhibited a high Pearson correlation coefficient of 0.78 with LysoTracker Red, indicating predominant lysosomal localization in the early stage of cellular uptake. This specific localization allowed lysosomal esterase to hydrolyze the ester bonds of ACEF and expose the EGTA moiety for subsequent Ca^2+^ capture. By 2 h post‐incubation, the Pearson correlation coefficient between ACEF‐FITC and LysoTracker Red decreased significantly to 0.28, and the ACEF‐FITC signals dispersed more broadly in the cytoplasmic region, indicating progressive intracellular redistribution after lysosomal processing. Notably, the Pearson correlation coefficients between ACEF‐FITC and MitoTracker Red remained below 0.3 at both time points, confirming weak colocalization between ACEF and mitochondria. To further characterize the intracellular trafficking process of ACEF, time‐dependent lysosomal escape analysis was performed in 4T1 cells from 0.5 to 8 h. As shown in Figure S27a, ACEF‐FITC initially showed strong colocalization with LysoTracker Red, indicating predominant lysosomal localization at the early stage of cellular uptake. With prolonged incubation, the green ACEF‐FITC signal gradually separated from the red lysosomal signal, suggesting progressive lysosomal escape and intracellular redistribution of ACEF. Quantitative analysis based on the Manders’ overlap coefficient further confirmed this trend, showing a time‐dependent increase in lysosomal escape efficiency, which approached approximately 80% at 8 h (Figure S27c). These results indicate that ACEF was not persistently confined within lysosomes after endocytosis, but could effectively escape from lysosomal compartments to support its subsequent intracellular function. To further correlate lysosomal escape with intracellular Ca^2+^ regulation, a time‐dependent intracellular Ca^2+^ fluorescence was analyzed using Fluo‐4 AM staining and flow cytometry. 4T1 cells were treated with ACEF for 0, 0.5, 1, 2, 4, 6, and 8 h, followed by Fluo‐4 AM staining. As shown in Figure S27b,c, the intracellular Ca^2+^‐associated Fluo‐4 fluorescence gradually decreased with prolonged incubation time, showing an opposite trend to the increasing lysosomal escape efficiency. At early time points, when ACEF was mainly retained in lysosomes, the intracellular Ca^2+^ signal remained relatively high. After longer incubation, increased lysosomal escape and intracellular redistribution of ACEF were accompanied by a marked decrease in Ca^2+^ fluorescence. This time‐correlated relationship suggests that ACEF‐mediated Ca^2+^ regulation is closely associated with intracellular delivery and lysosomal escape, making nonspecific extracellular EGTA leakage unlikely to be the major cause of the observed Ca^2+^ decrease. Given the important role of Ca^2+^ in regulating intracellular signaling [54, 55], the Ca^2+^ capture capability of ACEF in 4T1 cells was further evaluated. As shown in Figure 4c, PBS, free Au+CeO_2_, AC, and ACF (without EGTA) did not significantly reduce intracellular Ca^2+^ levels. In contrast, ACE, ACEF, and ACEF+NIR all markedly reduced intracellular Ca^2+^ levels, with ACEF showing stronger activity than ACE, consistent with the contribution of FA‐mediated cellular uptake. These results indicate that EGTA is the key functional unit for Ca^2+^ capture, while FA enhances this effect by improving tumor‐cell uptake. To further mimic the intracellularly relevant environment after cellular processing, ACEF was incubated in 4T1 cell lysate and analyzed by UV–vis spectroscopy, DLS, and photothermal testing (Figure S28). Aggregation‐associated optical, size, and photothermal changes were observed, which showed that ACEF could indeed undergo aggregation‐associated activation in the intracellular environment of the tumor, further supporting that the endogenous Ca^2+^ is sufficient to induce the intracellular therapeutic processes of ACEF.

The decrease in intracellular Ca^2+^ concentration would disrupt the mitochondrial Ca^2+^ transport process, causing mitochondrial dysfunction and a reduction in membrane potential, thereby disrupting normal cellular physiological activities. Thus, a potential‐dependent JC‐1 fluorescent probe was employed to detect the impact of Ca^2+^ capture on mitochondrial membrane potential. In mitochondria with normal membrane potential, the JC‐1 probe aggregates into polymers within the mitochondrial matrix, resulting in red fluorescence emission. Conversely, in mitochondria with diminished membrane potential, JC‐1 remains in its monomeric form and emits green fluorescence. As seen from Figure 4d, the 4T1 cells treated with PBS, free Au+CeO_2_, AC, and ACF groups displayed strong red fluorescence and minimal green fluorescence, indicating that their mitochondrial membrane potentials were almost unaffected. In contrast, cells treated with ACE, ACEF, and ACEF+NIR exhibited obviously weakened red fluorescence accompanied by markedly enhanced green fluorescence compared to the other groups, suggesting a decline in mitochondrial membrane potential. Subsequently, quantitative analysis of the JC‐1 staining by flow cytometry yielded results consistent with the above fluorescence microscope observations (Figure 4e). Collectively, these findings indicated that only nanoparticles with Ca^2+^ trapping capability could induce mitochondrial damage and cause a decrease in membrane potential in cancer cells.

Meanwhile, to verify the synergistic anti‐tumor mechanism, ROS production (a key CDT factor) was detected by DCFH‐DA probe (Figure 4f, g). ROS generation was observed in all groups except PBS. The ACEF+NIR group exhibited the most robust ROS production, followed by the ACE+NIR group. The ACEF without NIR and the ACE groups showed moderate ROS levels, which were higher than those of the ACF group and the free Au+CeO_2_. This indicated that ROS generation was dependent on the Ce valence state cycling induced by NIR (Ce^4+^ to Ce^3+^), the enhanced EGTA accumulation, and the FA‐mediated targeting. To further evaluate whether ACEF NPs might interfere with macrophage‐associated inflammatory activation after tumor treatment, macrophage polarization experiments were performed under different microenvironmental conditions. RAW264.7 macrophages were cultured with conditioned medium collected from treated 4T1 cells, whereas THP‐1‐derived macrophages were stimulated with LPS + IFN‐γ, followed by flow cytometry analysis of CD86 expression (Figure S29). Under acidic conditions (pH 6.8), ACEF did not cause an obvious reduction in the CD86‐positive population, whereas under neutral conditions (pH 7.4), a reduced CD86‐positive population was observed. These results further support that ACEF preferentially preserves pro‐inflammatory activation under acidic conditions while alleviating excessive inflammatory activation under neutral conditions.

To verify the tumor‐specific photothermal‐chemical kinetic synergism of ACEF nanoparticles, the cellular activity of MCF‐10A and 4T1 cells in different treatment groups was evaluated by MTT assay (Figure 4h). The free Au+CeO_2_ mixture showed the weakest anti‐tumor activity (11.5% cell death), with negligible improvement after NIR irradiation (11.7%), confirming the necessity of the Au@CeO_2_ hybrid nanoparticles structure. The AC group (lacking EGTA) had a cell death rate of 23.3% without NIR, increasing to 36.8% with NIR, reflecting the basic efficacy of the Au@CeO_2_ core and mild photothermal enhancement. The ACF group (ACEF without EGTA) achieved 39.7% cell death without NIR, and NIR only slightly elevated the cell death rate to 39.9%, indicating FA‐mediated targeting improves therapeutic efficacy but lacks NIR synergy due to the absence of EGTA. The ACE group (ACEF without FA) showed 54.3% cell death without NIR (up to 62.7% with NIR), verifying the critical role of EGTA‐mediated Ca^2+^ capture and its synergy with photothermal therapy. Notably, ACEF (with FA and EGTA) reached 69.9% cell death without NIR, and this drastically increased to 87.5% with NIR, confirming the synergistic amplification of CDT/PTT by FA targeting, Ca^2+^ capture, and NIR activation. Importantly, ACEF+NIR showed negligible toxicity (< 10%) to MCF‐10A cells, demonstrating tumor‐selective therapy via TME‐specific activation (Figure 4i). To further clarify the contribution of different therapeutic modules, MTT assays were performed using PBS, AEF, AEF+NIR, ACF, and ACEF+NIR groups (Figure S30). AEF was used to evaluate the Ca^2+^ capture module, AEF+NIR was used to evaluate the Ca^2+^ capture‐induced aggregation/photothermal module, and ACF was used to evaluate the CDT‐related module. Compared with these module‐control groups, the complete ACEF+NIR system showed the strongest cytotoxicity. Notably, the therapeutic effect of ACEF+NIR was stronger than that expected from either individual module alone, supporting the cooperative amplification among Ca^2+^ capture, aggregation‐enhanced PTT, and CeO_2_‐related CDT.

Excessive accumulation of ROS impairs mitochondrial membrane potential and activates apoptotic pathways, eventually inducing cancer cell death. To quantitatively evaluate the apoptotic effect of each group on 4T1 cells, Annexin V‐FITC/PI double‐staining flow cytometry was performed (Figure 4j). After 24 h of treatment, the apoptosis rate (including early and late apoptosis) was quantified, and a clear gradient was observed among all groups. The PBS group had the lowest apoptosis rate (7.13%), indicating minimal spontaneous cell death. The free Au+CeO_2_ mixture showed a slightly increased apoptosis rate (11.77%), consistent with its weak anti‐tumor activity. In contrast, the AC (lacking EGTA) exhibited an apoptosis rate of 24.91%, reflecting the basic anti‐tumor activity of the Au@CeO_2_ core. The ACF (ACEF without EGTA) achieved an apoptosis rate of 31.61%, which was higher than that of the AC group due to FA‐mediated tumor targeting but lower than that of the EGTA‐containing groups, confirming the lack of Ca^2+^ response‐related apoptotic enhancement. The ACE group (ACEF without FA) achieved an apoptosis rate of 35.74%, verifying the contribution of EGTA‐mediated Ca^2+^ capture to inducing cell apoptosis. The ACEF group (without NIR irradiation) reached an apoptosis rate of approximately 41.01%, benefiting from the synergistic effect of FA targeting and Ca^2+^ capture. Notably, the ACEF+NIR group showed the highest apoptosis rate (55.40%), reaching the highest apoptosis rate, confirming that NIR‐triggered PTT/CDT synergy can efficiently amplify tumor cell apoptosis.

Metastasis is a hallmark of cancer and the primary cause of mortality in patients. Since Ca^2+^‐dependent signaling pathways can promote tumor cell proliferation and migration, the effect of Ca^2+^ depletion on cell metastatic potential was investigated to assess the anti‐metastatic activity of ACEF. As displayed in Figure 4k–n, the PBS, free Au+CeO_2_, AC, and ACF groups showed nearly complete scratch healing in 48 h, while groups with EGTA (ACE, ACEF, ACEF+NIR) significantly reduced migration distance and cell number, with ACEF+NIR showing the strongest inhibition. Similar results were observed in transwell assays, confirming that Ca^2+^ capture (mediated by EGTA) was the core of anti‐metastatic effects, while FA‐mediated targeting and NIR activation provide additive benefits.

Generally, ACEF utilized the enhanced POD‐like activity of the Au@CeO_2_ hybrid nanoparticles structure, FA‐mediated tumor targeting, EGTA‐dependent Ca^2+^ capture in response to TME, and the Ce valence state cycling enhanced CDT and photothermal therapy (PTT) triggered by near‐infrared light, achieving precise tumor elimination and metastasis inhibition, providing a multifunctional platform for precision oncology.

### 2.5 In Vivo Antitumor Activities of ACEF

**FIGURE 5:**
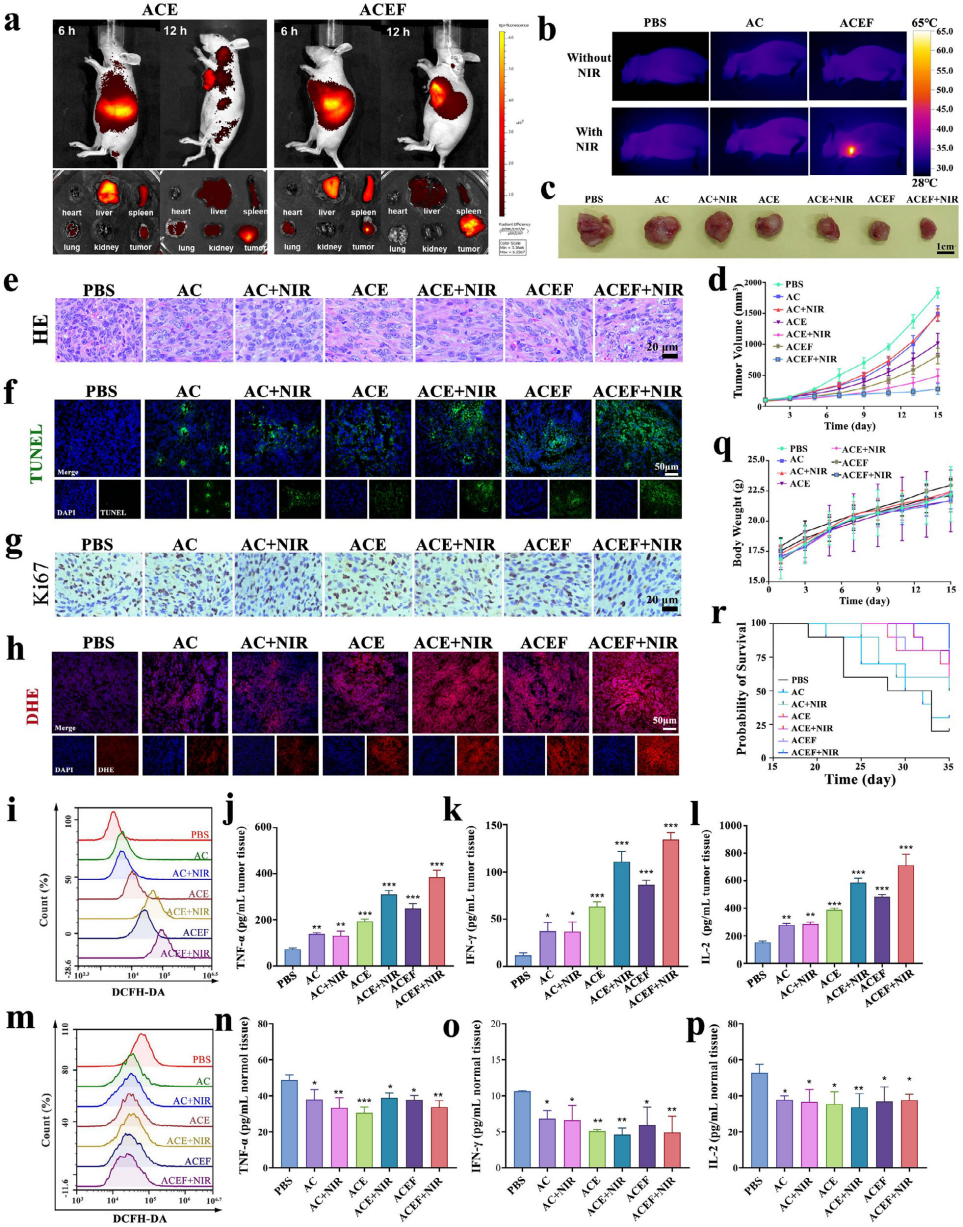
(a) In vivo fluorescence images of the tumor‐bearing mice at 6 h and 12 h after injection of ACE (nanoparticle without FA targeting) and ACEF (nanoparticle with FA targeting). (b) The photothermal images of tumor‐bearing mice after injection of PBS, AC, and ACEF with and without 660 nm irradiation. (c) Photos of excised tumors from mice treated with various formulas on the 15 th day. (d) Tumor volume curves over time following various treatments. Representative images of (e) H&E staining, (f) TUNEL assay, (g) Ki67 immunohistochemistry, and (h) DHE staining in tumor tissues. (i) ROS levels, and (j) TNF‐𝛼, (k) IFN‐γ, and (l) IL‐2 concentrations in tumor tissues (n = 3). (m) ROS levels, and (n) TNF‐𝛼, (o) IFN‐γ, and (p) IL‐2 concentrations in surrounding normal tissues after different treatments (n = 3). (q) Body weight of mice over time (n = 3). (r) Survival curves of mice in different treatment groups (n = 10). Error bars represent mean ± SD. (^***^ *p* < 0.001, ^**^ *p* < 0.01, ^*^ *p* < 0.05).

Building upon the in vitro validation of TME‐activated therapeutic synergy, we further evaluated the tumor‐targeting capability and in vivo antitumor efficacy of ACEF NPs in 4T1 tumor‐bearing mice. DiR‐labeled ACE and ACEF NPs were intravenously administered for dynamic biodistribution tracking via real‐time fluorescence imaging. Herein, the ACE NPs entered tumor tissues only through the enhanced permeability and retention (EPR) effect, whereas the ACEF NPs achieved tumor accumulation through the synergistic effect of the EPR effect and folate receptor‐mediated tumor‐targeting. As seen from Figure 5a, fluorescence intensity in tumor sites of both groups gradually increased over time. However, the fluorescence signal in the DiR‐ACEF NP groups was markedly stronger than that in the DiR‐ACE NPs group, suggesting that the ACEF NPs exerted superior tumor‐selective delivery efficiency due to FA modification, and these nanozymes had prolonged retention in the tumor for sustained therapy. Quantitative analysis of the fluorescence intensity in tumor sites revealed that the tumor fluorescence intensity of ACEF NPs was 1.4‐fold higher than that of ACE NPs at 12 h post‐administration (Figure S31), further confirming the tumor‐targeting capability of ACEF.

This tumor‐selective accumulation was further amplified under NIR irradiation. As shown in Figure 5b, the ACEF+NIR group achieved localized hyperthermia (55.3°C) through photothermal conversion combined with Ca^2+^‐chelation‐enhanced NP retention, whereas the AC group (lacking EGTA) failed to generate substantial temperature elevation (<40°C), ​confirming that Ca^2+^‐dependent aggregation was critical for PTT. To directly monitor the synchronicity of the “aggregation‐photothermal activation” process in vivo, ACF and ACEF were modified with aggregation‐induced emission (AIE) molecules, and were intravenously administered into tumor‐bearing mice. At 0, 6 h, 12 h, and 24 h post‐administration, the tumor regions were irradiated with NIR light, and temperature changes were recorded using an infrared thermal imager. Tumors were then harvested, and AIE fluorescence intensity was observed via fluorescence microscopy to assess nanoparticle aggregation. As shown in Figure S32a, the tumor temperature in the ACEF group increased in a time‐dependent manner, gradually rising at 6 and 12 h and peaking at 24 h, whereas the ACF group exhibited minimal temperature changes across all time points. The AIE fluorescence intensity in tumor tissues closely mirrored the temperature changes (Figure S32b,c). The ACEF‐treated tumors showed a gradual increase in fluorescence intensity from 6 to 24 h, with the strongest fluorescence at 24 h (consistent with peak aggregation), whereas the ACF‐treated tumors displayed weak fluorescence across all time points. These findings confirmed that ACEF could specifically accumulate and aggregate within tumors in a time‐dependent manner, and its aggregation degree was positively correlated with photothermal activity. The gradual aggregation from 6 to 24 h led to progressive enhancement of photothermal effects, and peak aggregation at 24 h corresponded to the strongest photothermal activity. In contrast, ACF, lacking EGTA, failed to aggregate effectively, resulting in negligible photothermal effects.

To quantify the therapeutic outcomes, 4T1 tumor‐bearing mice were randomly divided into seven groups and treated with PBS, AC, AC+NIR, ACE, ACE+NIR, ACEF, and ACEF+NIR. Tumor volumes were monitored over a period of 15 days (Figure 5d), then the final tumor tissues were collected, photographed (Figure 5c), and weighed (Figure S33). It was found that AC and AC+NIR exhibited weak antitumor properties, which could be attributed to the peroxide properties of CeO_2,_ leading to ROS overload in tumor tissues. EGTA modification (ACE) further enhanced tumor inhibition through Ca^2+^ chelation, and the combination with NIR irradiation (ACE+NIR) produced additional efficacy by PTT activation. Notably, ACEF+NIR showed the most pronounced inhibition of tumor growth, attributable to the synergistic effects of FA‐mediated targeting, Ca^2+^‐enhanced retention, and dual ROS/PTT activation. Hematoxylin and eosin (H&E) staining (Figure 5e) revealed distinct pathological changes among different groups. Tumors from the PBS group exhibited a dense, well‐preserved cellular structure with large, round nuclei and abundant cytoplasm, indicative of active proliferation. In contrast, the AC and AC+NIR groups showed moderate tumor cell damage, characterized by occasional cell shrinkage and karyorrhexis. The ACE and ACE+NIR groups displayed more extensive areas of necrosis and vacuolization, suggesting enhanced cytotoxicity. Most strikingly, the ACEF+NIR group presented the most severe tumor destruction, with large regions of necrosis and loss of cellular architecture, confirming the potent synergistic antitumor effect.

Tumor cell apoptosis was assessed via TUNEL staining (Figure 5f). The ACEF+NIR group exhibited the highest capacity to induce apoptosis, with a significant increase in TUNEL‐positive cells (green fluorescence) compared to other groups, which was consistent with the pronounced tumor inhibition observed earlier. Proliferative activity was assessed by Ki67 immunohistochemistry (Figure 5g). The PBS group showed a high Ki67 labeling index, reflecting robust tumor cell proliferation. The AC and ACE groups demonstrated a moderate decrease in Ki67‐positive cells, while the ACEF group further reduced the index due to improved tumor targeting. The ACEF+NIR group exhibited the lowest Ki67 expression, with only scattered positive cells, directly correlating with the massive cell death observed in H&E staining and the high apoptosis rate in TUNEL staining. In vivo ROS production, a key indicator of CDT efficacy, was detected via DHE staining (Figure 5h). A clear gradient in superoxide anion fluorescence intensity was observed, ranking from highest in the ACEF+NIR group, followed by the ACEF group, ACE group, AC group, and PBS group. This gradient was attributed to the gradual enhancement of peroxidase mimetic activity. AC (Au@CeO_2_) possesses basic peroxidase properties; ACE achieves local tumor aggregation via Ca^2+^ chelation, concentrating peroxidase activity; ACEF enhances tumor targeting through FA for deeper accumulation; ACEF+NIR not only introduces PTT but also boosts POD‐like activity through NIR‐associated Ce valence‐state regeneration, thereby generating the highest level of ROS. To quantitatively validate this observation and exclude off‐target ROS generation, ROS levels in both tumor and normal tissues were systematically assessed by flow cytometry. Consistent with the DHE staining results, ACEF+NIR induced robust ROS production in tumors (Figure 5i and Figure S34), while ROS levels in normal organs remained unaffected (Figure 5m and Figure S35). This tumor‐specific ROS elevation was attributed to the TME‐triggered POD‐like activity of CeO_2_, which simultaneously scavenged leaked ROS in healthy tissues via its CAT‐like and SOD‐like functions. ELISA quantification of cytokines in tumor and adjacent normal tissues (TNF‐α, IFN‐γ, IL‐2) confirmed the spatial specificity of ACEF. Pro‐inflammatory factors showed significant elevation in tumor tissues (Figure 5j–l), consistent with immunogenic cell death activation, while maintaining baseline levels in adjacent normal tissues (Figure 5n–p). This tumor‐selective cytokine profile demonstrated the potential of ACEF for precise therapeutic targeting while preserving healthy tissue homeostasis.

To further evaluate the immunological effects of ACEF in tumor and healthy tissues, flow cytometry was performed to quantify immune cell populations in both tumor tissues and adjacent normal tissues. In tumor tissues, ACEF treatment decreased the proportion of CD206^+^ M2‐like macrophages and increased CD86^+^ M1‐like macrophages (Figure S36), indicating a shift from an immunosuppressive macrophage phenotype toward an anti‐tumor inflammatory phenotype. Moreover, the proportions of mature dendritic cells (CD11c^+^ MHC‐II^+^) and cytotoxic CD8^+^ T cells were also increased in the tumor region, suggesting activation of the antigen‐presentation‐related anti‐tumor immune cascade. In adjacent normal tissues, ACEF did not induce abnormal immune activation, as no obvious increase in CD86^+^ M1‐like macrophages, mature dendritic cells, or CD8^+^ T cells was observed (Figure S37). Importantly, these immune cell populations were not reduced compared with the control group, indicating that ACEF did not suppress the basal immune status of healthy tissues. Meanwhile, the proportion of CD206^+^ macrophages was slightly elevated in normal tissues, which may be associated with immune tolerance and tissue repair. These results suggest that ACEF can promote anti‐tumor immune activation in tumor tissues while avoiding both excessive inflammatory activation and immune suppression in adjacent normal tissues.

To comprehensively evaluate the biosafety effect of ACEF, systemic toxicity and long‐term survival outcomes were monitored. No significant body weight loss or behavioral abnormalities were observed in any treatment groups throughout the study (Figure 5q), indicating minimal acute toxicity. The effective anti‐tumor mechanism, together with the safety of the treatment process, significantly prolonged the survival of tumor‐bearing mice, suggesting that our nanoparticles could be a promising therapeutic platform for malignant tumors (Figure 5r).

**FIGURE 6:**
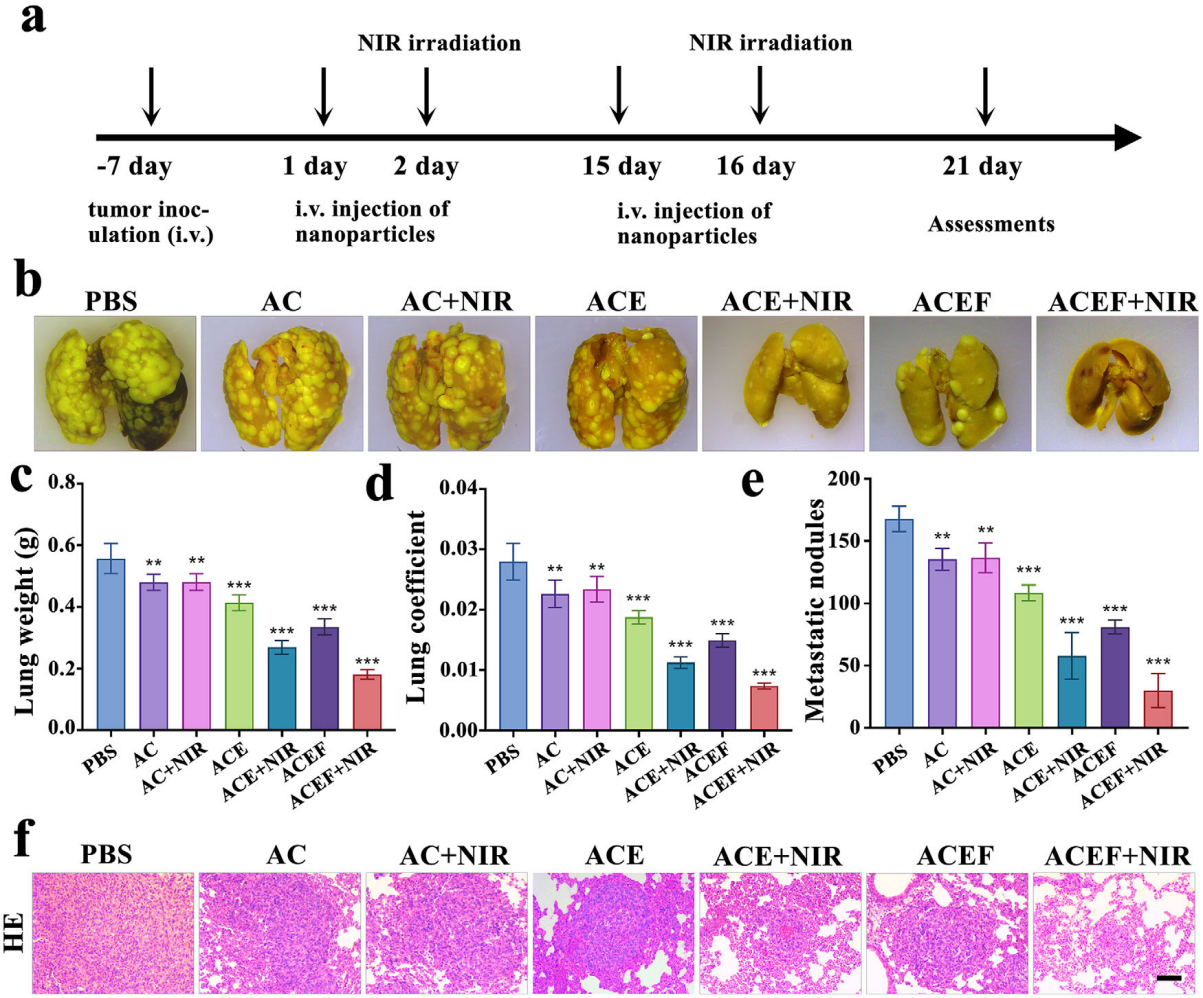
(a) Therapeutic schedule for 4T1 metastatic tumor‐bearing mice. (b) Representative images of lung metastases obtained from tumor‐bearing mice after different treatments on the 21st day. (c) Lung weight and (d) Lung coefficient of those lung samples from different treatments on the 21st day. (e) Number of metastatic nodules in lung samples from different groups. (f) H&E staining images of the lungs obtained from tumor‐bearing mice from the various groups. The scale bar is 100 µm. Error bars represent mean ± standard deviation (n = 5). (^***^ *p* < 0.001, ^**^ *p* < 0.01).

Besides directly inhibiting primary tumor growth, ACEF was expected to effectively impede tumor cell metastasis through Ca^2+^ capture. The lung metastasis mouse model was established, and the lungs from treated mice were collected for histological evaluation (Figure 6a). As shown in Figure 6b, numerous metastatic nodules were observed in the PBS group, and the number of metastatic nodules gradually decreased after different treatments. The ACEF+NIR group exhibited the most pronounced suppression of lung metastasis. Quantitative analyses of lung weight (Figure 6c), lung coefficient (Figure 6d), and metastatic nodules (Figure 6e) also proved this point. H&E staining further confirmed the inhibition of tumor metastasis, revealing well‐preserved lung architecture in the ACEF+NIR group compared with other groups (Figure 6f).

To clarify the in vivo fate of ACEF and ensure its biosafety for antitumor applications, we tracked its clearance, excretion pathways, and biodistribution using inductively coupled plasma‐mass spectrometry, by quantifying Au content (as a tracer for ACEF) in blood, urine, feces, and major organs at specific time points post‐administration. As shown in Figure S38a, the blood concentration‐time curve showed a biphasic decline and was fitted using a two‐compartment model, giving a distribution half‐life (t_1/2α_) of approximately 0.58 h and an elimination half‐life (t_1/2β_) of 14.44 h. Moreover, the biodistribution of ACEF at 24 h post‐administration (Figure S38b) showed predominant accumulation in the liver and spleen, which was a common phenomenon attributed to RES clearance, with minimal accumulation in the lungs, kidneys, and heart. This low off‐target accumulation in vital organs, coupled with efficient hepatobiliary excretion, further validated the favorable biosafety profile of ACEF for in vivo antitumor therapy. Figure S38c,d further demonstrated that ACEF was primarily eliminated via the hepatobiliary pathway within 60 h, with about 60% of the administered dose excreted through feces, while urinary excretion was less than 1%. The metabolic pattern was consistent with the size and surface properties of nanoparticles prone to RES recognition.

To further evaluate the stability and possible off‐target activation of ACEF in blood and major organs, ACEF was first incubated with fresh mouse serum for 24 h, followed by UV–vis and photothermal analyses. As shown in Figure S39, ACEF did not exhibit obvious aggregation‐associated redshift or photothermal activation after serum incubation, indicating that ACEF is in a dormant and safe state under serum conditions. To further examine the possible state of ACEF in major organs, ACEF was incubated with lysates from the liver, spleen, lung, and kidney, followed by UV–vis and photothermal analyses (Figure S40). Among these tested organ‐associated *ex vivo* conditions, only the liver lysate induced a redshift tendency and a slight increase in photothermal temperature, whereas the effect remained markedly weaker than that observed in the 4T1 cell lysate.

To evaluate the systemic toxicity of ACEF, mouse blood samples were collected before injection, as well as at 1, 7, and 14 days post‐administration, for routine blood tests, liver and kidney function biochemical assays, and serum inflammatory cytokine detection. Routine blood parameters (Figure S41), including white blood cell count (WBC), lymphocyte count (Lymph#), granulocyte count (Gran#), red blood cell count (RBC), platelet count (PLT), and hemoglobin (HGB), showed no obvious abnormal fluctuations at 1, 7, and 14 days post‐injection compared with the pre‐injection baseline. Considering the Ca^2+^‐chelating property of EGTA, serum Ca^2+^ concentration, coagulation function, and representative serum metal/divalent ion levels were further evaluated before ACEF administration and at 30 min and 24 h post‐administration. As shown in Figure S42, no obvious decrease in serum Ca^2+^ concentration was detected after ACEF administration. Coagulation function was assessed using prothrombin time (PT), activated partial thromboplastin time (APTT), thrombin time (TT), and fibrinogen (FIB), and no obvious abnormalities were observed at the tested time points (Figure S43). Meanwhile, representative serum metal/divalent ion levels, including Mg^2+^, Zn^2+^, and Fe^2+^, remained stable after ACEF administration (Figure S44). These results indicate that ACEF did not measurably disturb systemic Ca^2+^ homeostasis, coagulation function, or representative serum metal ion levels under the tested conditions. In addition, no obvious fluctuations were observed in liver and kidney function‐related biochemical indicators (Figure S45) among all groups, including alanine aminotransferase (ALT), aspartate aminotransferase (AST), creatinine (CREA), total bilirubin (TBIL), and urea (UREA), suggesting the absence of hepatic or renal toxicity.

Serum inflammatory cytokines, including tumor necrosis factor‐α (TNF‐α), interleukin‐6 (IL‐6), and interferon‐γ (IFN‐γ), were quantified at the same time points (Figure S46). No significant elevation or abnormal changes were detected in the ACEF‐treated group compared with the baseline, indicating the absence of systemic inflammatory responses induced by ACEF. Furthermore, histological analysis of inflammatory infiltration was performed on the heart, liver, spleen, lung, and kidney of ACEF‐treated mice using immunohistochemical staining for inflammatory cell markers (Figure S47). The infiltration of CD45^+^ (pan‐leukocyte marker), F4/80^+^ (macrophage marker), Ly6G^+^ (neutrophil marker), and CD3^+^ (T cell marker) cells exhibited no significant difference compared with the baseline. Meanwhile, the organ coefficients of the heart, liver, spleen, lung, and kidney in ACEF‐treated mice were normal (Figure S48), and no obvious toxic lesions or tissue damage were detected in H&E staining results (Figure S49).

Generally, ACEF demonstrated dual therapeutic efficacy against both primary and metastatic tumors through TME‐responsive activation. The FA‐mediated active targeting, combined with EGTA‐induced Ca^2+^ capture, ensured the preferential accumulation and retention of ACEF at tumor sites. This spatiotemporal confinement enabled localized hyperthermia and catalytic ROS generation under NIR irradiation, leading to the potent eradication of primary tumors. ​Notably, the Ca^2+^‐depleting strategy further disrupted metastatic dissemination, significantly suppressing pulmonary metastasis without systemic toxicity. The self‐limiting therapeutic activation within tumors, coupled with the intrinsic ROS‐scavenging capacity in normal tissues, validated ACEF as a precision nanoplatform for the synergistic primary and metastatic tumors.

## 3 Conclusion

In summary, a microenvironment‐adaptive Au@CeO_2_ hybrid nanoparticle (ACEF) was developed for tumor‐biased photothermal/chemodynamic therapy and ROS regulation in surrounding normal tissues. Under tumor‐associated conditions, esterase/Ca^2+^‐ medicated activation promoted nanoparticle aggregation, thereby enhancing NIR‐responsive photothermal conversion and ROS‐generating catalytic activity. In contrast, under neutral physiological conditions, dispersed ACEF mainly showed ROS‐scavenging behavior, helping reduce excessive oxidative/inflammatory stress outside tumor regions. In vitro and in vivo results demonstrated that ACEF effectively inhibited primary tumor growth and lung metastasis, while no obvious systemic toxicity or major organ injury was observed under the tested conditions. Nevertheless, further studies are still needed to directly monitor NIR‐associated interfacial electron/valence‐state changes in complex biological environments, as well as to evaluate long‐term biosafety, immune effects, and therapeutic performance in more clinically relevant models. Overall, this work provides a microenvironment‐adaptive strategy for balancing localized tumor therapy with off‐target inflammation regulation.

## Author Contributions

**Wenyun Mu**: data curation, formal analysis, investigation, writing – original draft, project administration. **Wenjuan Tang**: data curation, formal analysis, investigation, writing – original draft. **Handan Zhang**: data curation, formal analysis, investigation. **Jie Liu**: data curation, formal analysis. **Jiaqi Zhang**: data curation, investigation. **Yu Yao**: writing – original draft. **Xiao Fu**: data curation. **Xin Chen**: conceptualization, project administration, supervision, funding acquisition, writing – review and editing. **Yanmin Zhang**: supervision, project administration, writing, review and editing, and funding acquisition.

## Conflicts of Interest

The authors declare no conflict of interest.

## Supporting information




**Supporting File**: advs76172‐sup‐0001‐SuppMat.docx.

## Data Availability

The data that support the findings of this study are available from the corresponding author upon reasonable request.
