# Supplementary material for: Engineered Au@CeO2 Hybrid Nanoparticles With Microenvironment Dependent Self‐Adjustability for Integration of Tumor‐Specific Photothermal‐Chemodynamic Therapy and Inflammation Prevention
Source: Adv Sci (Weinh). 2026 Jun 22;13(51):e76172. doi: 10.1002/advs.76172 (PMC13337052; doi:10.1002/advs.76172)
Supplement: Supplementary file 1 [file ADVS-13-e76172-s001.docx]

**Supporting information**

**for**

**Engineered Au@CeO_2_ Hybrid Nanoparticles with Microenvironment Dependent Self-adjustability for Integration of Tumor-specific Photothermal-Chemodynamic Therapy and Inflammation Prevention**

Wenyun Mu ^a,b #^, Wenjuan Tang ^a,c,d #^, Handan Zhang ^b, #^, Jie Liu ^b^, Jiaqi Zhang ^d^, Yu Yao ^a^, Xiao Fu ^a^, Xin Chen ^b, *^, Yanmin Zhang ^a,d *^

*^a^ Department of Medical Oncology, The First Affiliated Hospital of Xi'an Jiaotong University, Xi'an, Shaanxi, 710061 P.R. China*

*^b^ Department of Chemical Engineering, Shaanxi Key Laboratory of Energy Chemical Process Intensification, Institution of Polymer Science in Chemical Engineering, School of Chemical Engineering and Technology, Xi’an Jiaotong University, Xi’an 710049, P.R. China*

*^c^ Department of Gastroenterology, The First Affiliated Hospital of Xi'an Jiaotong University, Xi'an, Shaanxi, 710061, P.R. China*

*^d^ School of Pharmacy, Health Science Center, Xi’an Jiaotong University, Xi’an, 710061, P.R. China*

^#^ These authors contributed equally to this work.

^*^ Corresponding Authors (E-mail: [chenx2015@xjtu.edu.cn](mailto:chenx2015@xjtu.edu.cn,); zhang2008@xjtu.edu.cn)

## 1. **Materials**

Ethylene glycol tetraacetic acid (EGTA), N-hydroxysuccinimide (NHS), 1-(3-dimethylaminopropyl)-3-ethylcarbodiimide hydrochloride (EDC·HCl), 3,3',5,5'-tetramethylbenzidine (TMB) were purchased from Macklin (Shanghai, China); HO-PEG5000-Br was purchased from Ponsure (Shanghai, China); Sodium hydroxide (NaOH), trisodium citrate, sodium chloride (NaCl) were purchased from Damao (Tianjin, China); hydrogen peroxide (H_2_O_2_) was purchased from Hengxing (Tianjin, China). Cerium acetate, potassium chloroplatinate (K_2_PtCl_4_), chloroauric acid trihydrate (HAuCl_4_·3H_2_O), folic acid (FA) were purchased from Aladdin (Shanghai, China); Dulbecco’s modified Eagle’s medium (DMEM), and fetal bovine serum were obtained from Gibco (Grand Island, NY, USA). Mitochondrial membrane potential assay kit with JC-1 and 2’,7’-dichlorofluorescein diacetate (DCFH-DA) were purchased from Beyotime (Shanghai, China).

## **2. Characterizations.**

The chemical structure of the nanoparticles was characterized using a Fourier infrared spectrometer (Nicolet iS50). The ^1^H-spectra of the MEA-EGTA-PEG-FA was determined by a 400 MHz JEOL nuclear magnetic resonance spectrometer (NMR, JNM-ECZ400S/L1). The particle size and zeta potential of the nanoparticles were determined by a Malvern Zetasizer (ZSE) with dynamic light scattering (DLS) equipment. Ultraviolet-visible (UV-vis) spectra were recorded by a UV-vis spectrophotometer (Thermo Scientific, GENESYS 150). Fluorescence spectra were measured using a fluorescence spectrometer (GangDong, F-280). The photothermal capacity of the materials was measured using a near-infrared laser light source (660NL-2W) and a thermal imager (Testo AG ATS024T-W050V). The morphology of the nanoparticles was observed using a Lorenz Transmission Electron Microscope (TEM) (Talos F200X with Energy Dispersive Spectrometer). The X-ray photoelectron spectra (XPS) of the nanoparticles were recorded with an X-ray photoelectron spectrometer (XPS) (ThermoFisher Scientific ESCALAB Xi+).

## **3. Synthesis of MEA-EGTA-PEG-FA**

Referring to the previous work of our lab, MEA-EGTA-PEG-FA molecules were synthesized through a two-step substitution reaction, with detailed procedures and key parameters as follows: All glassware and magnetic stir bars used in the experiment were first soaked in piranha solution (concentrated H_2_SO_4_:30% H_2_O_2_ = 3:1, v/v) for 2 h to remove organic contaminants, then thoroughly rinsed with ultra-pure water and dried under nitrogen atmosphere for later use. For the first step of conjugation, 19 mg of EGTA and 8.0 mg of sodium hydroxide were dissolved in 20 mL of ultra-pure water and stirred until completely dissolved. Next, 21 mg of EDC (1-ethyl-3-(3-dimethylaminopropyl)carbodiimide hydrochloride) and 6.7 mg of NHS (N-hydroxysuccinimide) were accurately weighed and added to the EGTA solution, followed by stirring at room temperature (25 ± 1 °C) for 30 min to activate the carboxyl groups of EGTA; the pH value of the system was adjusted to 5.5 with 0.1 M HCl/NaOH solution to obtain Mixed Solution 1. Subsequently, 3.9 mg of 2-mercaptoethylamine (MEA), 250 mg of HO-PEG_5000_-Br, and 23 μL of triethylamine (serving as a catalyst to promote the reaction) were sequentially added to Mixed Solution 1, and the reaction system was stirred at room temperature (25 ± 1 °C) in the dark for 48 h to avoid photodegradation of reactive groups. After the reaction, the mixture was transferred to a dialysis bag (molecular weight cutoff, MWCO = 3500 Da) and dialyzed against 1500 mL of ultra-pure water for 24 h (dialysate replaced every 8 h) to remove unreacted EDC, NHS, and excess MEA, yielding Mixed Solution 2. For the second step of conjugation to obtain the final product, 33 mg of folic acid (FA) was dissolved in 10 mL of ultra-pure water, then 63 mg of EDC and 20 mg of NHS were added; the mixture was stirred at room temperature (25 ± 1 °C) in the dark for 30 min to activate the carboxyl groups of FA, obtaining Mixed Solution 3. Mixed Solution 3 was then slowly added dropwise to Mixed Solution 2, and the reaction was stirred at room temperature (25 ± 1 °C) in the dark for 48 h. After the reaction, the mixture was transferred to a dialysis bag (MWCO = 3500 Da) and dialyzed against 1500 mL of ultra-pure water for 24 h (dialysate replaced every 8 h) to remove unreacted FA and residual activating agents. The dialyzed solution was finally freeze-dried (temperature: -50 °C, pressure: 10 Pa) for 48 h to obtain MEA-EGTA-PEG-FA as a light yellow fluffy solid, with a total yield of 24 ± 5% (n = 3, calculated as the mass ratio of the final freeze-dried product to the total mass of initial EGTA, MEA, HO-PEG_5000_-Br, and FA).

## **4. Preparation of Au@CeO_2_ (AC) NPs**

Au NPs were prepared using the previous method. AuNPs were prepared by sodium citrate reduction method. First, 1000 mg of HAuCl_4_·3H_2_O was dissolved in 10 mL of ultra-pure water to obtain 0.254 M of the solution of HAuCl_4_·3H_2_O, which was stored in glass bottles at low temperature away from light. 88 mg sodium citrate was dissolved in 20 mL ultra-pure water to obtain 0.015 M sodium citrate solution. 200 mL of ultrapure water was added into a conical bottle, then add 220 μL of HAuCl_4_·3H_2_O (0.254 M), and heat on a 130 ℃ heating table until boiling, during which time continue to stir with magnetons. Immediately afterwards, 20 mL sodium citrate solution (0.015 M) was poured into the conical bottle, and the solution was boiled again and reacted for about 10 min. The solution changed from light yellow to wine red, indicating the formation of AuNPs.

The solution of 0.50 mM potassium chloroplatinite was obtained by dissolving 2 mg potassium chloroplatinite in 10 mL ultra-pure water. The solution of 10 mM cerium acetate was obtained by dissolving 31.7 mg cerium acetate in 10 mL ultra-pure water. Add 300 μL potassium chloroplatinite solution (0.50 mM) to 20 mL AuNPs solution and shake vigorously for 2 min. Then 500 μL cerium acetate solution (10 mM) was added and oscillated for 2 min. After reaction at room temperature for 24 h, Au@CeO_2_ nanoparticle solution was obtained.

## **5. Preparation of ACEF**

80 μL MEA-EGTA-PEG-FA solution was added into 20 mL Au@CeO_2_ solution and stirred at room temperature for 8 h to obtain ACEF hybrid nanoparticle solution.

## **6. In vitro peroxidase properties.**

The peroxidase activities of different nanoparticles were investigated spectrophotometrically in the presence of H_2_O_2_ using 3,3',5,5'-tetramethylbenzidine (TMB) as substrate. Briefly, H_2_O_2_ (2.5 mM) and TMB (800 µM) were mixed into different nanoparticles, and then the absorbance of the supernatant at 652 nm was recorded at different time points.

## **7. In vitro scavenging of ROS.**

The superoxide radicals (O_2_^−^·) scavenging capacity of different nanoparticles was assessed by the rate of inhibition of the nitroblue tetrazolium (NBT) photoreaction. Solutions of 0.4 mL of riboflavin (0.05 mM), 0.1 mL of methionine (125 mM), 0.1 mL of NBT (0.75 mM), and 0.4 mL of different nanoparticles were mixed in a PBS solution (pH = 7.4) to give a final solution volume of 2 mL. The resulting mixture was irradiated with ultraviolet (UV) light for 3 min, and the supernatant was recorded at 595 nm using a UV-Vis spectrophotometer. The control experiment was set up similarly, except that the nanoparticles were replaced with ultrapure water. The clearance was calculated as:

% O_2_^-^·scavenged = [1 - (AS – AS0)/(A1 - A0)]× 100%

AS0 and AS refer to the absorbance of the reaction mixture at 595 nm after the addition of nanomaterials before and after UV irradiation, respectively. A0 and A1 refer to the absorbance of the control solution before and after UV irradiation, respectively.

## **8. Photothermal conversion efficiency (PCE) calculation**

2 mL aliquot of each sample was added to a quartz cuvette. The sample was irradiated with 660 nm NIR laser (power density: 1.0 W/cm²) for 10 min, followed by 10 min of natural cooling. A thermocouple thermometer recorded the temperature every 30 s, with the ambient temperature maintained at 25 ± 1 °C. All measurements were performed in triplicate.

The photothermal conversion efficiency (η) was calculated using the standard formula:

$$\eta=\frac{hS\left( T_{max}-T_{surr} \right)-Q_{0}}{I(1-{10}^{-A_{660}})}$$

Where hS was derived from linear fitting of the cooling curve; T_max_-T_surr_ represents the maximum temperature difference between the sample and blank control; Q_0_ is the heat generated by DI water under laser irradiation; I is the laser power density; A_660_ is the sample absorbance at 660 nm, measured by a UV-Vis-NIR spectrophotometer. Data were expressed as mean ± SD (n = 3).

## **9. Cell Culture**

4T1 and MCF-10A cell lines were all obtained from the Shanghai Institute of Cell Biology at the Chinese Academy of Sciences. RPMI-1640 medium used in 4T1 cell culture is supplemented with 10% fetal bovine serum, 100 U/mL penicillin and 100 U/mL streptomycin. DMEM/F12 medium used in MCF-10A cell culture is supplemented with 5% horse serum, 10 μg/mL insulin, 20 ng/mL EGF, 100 ng/mL cholera toxin and 0.5 μg/mL hydrocortisone. Both cell lines were grown in an incubator with 5% CO_2_ at 37℃.

## **10. Cellular uptake**

4T1 cells and MCF-10A cells were seeded in 96 well-plates with 180 μL cell suspension per well for 24 h. Then, cells were treated with PBS, ACE or ACEF for 8 h. For fluorescence microscope study, cells were rinsed with PBS, fixed with 4% polyformaldehyde and stained with DAPI. images were captured by an inverted fluorescence microscope. For flow cytometry analysis, cells were collected, resuspended in PBS, and then analyzed using flow cytometry.

## **11. Cell viability assay（MTT）**

Cells were seeded in 96 well-plates with 180 μL cell suspension per well for 24 h. After treatment with different formulas, the cells were incubated for additional 48 h. Then the medium was replaced by serum-free medium with 10% MTT solution and incubated for 4–6 h. Subsequently DMSO was added to dissolve the formazan crystals, and absorbance was measured using Bio-Rad microplate reader.

## **12. Wound healing assay**

4T1 cells were first treated with the respective formulations for 48 h, then harvested and counted. The counted cells were seeded into 12-well plates. After the cells adhered, the medium was replaced by serum-free medium and a straight scratch was made in per well using a sterile P200 pipet tip. Images were captured at 0 h and 48 h using an inverted fluorescence microscope, and the wound closure rate was quantified.

## **13. Transwell migration assay**

4T1 cells were first treated with the respective formulations for 48 h, then harvested and counted. The counted cells were suspended in complete medium were cultured in the upper chamber, and the lower chamber was full of 500 µL complete medium. After the cells adhered, the upper medium was replaced with serum-free medium, and the lower medium was replaced with complete medium containing 20% FBS to induce migration. Following another 24 h incubation, non-migrated cells on the upper surface were removed carefully, while migrated cells on the lower surface were fixed with 4% polyformaldehyde and stained with 0.1% crystal violet. Images were acquired using an inverted fluorescence microscope.

## **14. Reactive oxygen species detection and mitochondrial membrane potential assay**

Intracellular ROS were measured by DCFH-DA probe, and mitochondrial membrane potential was analyzed with JC-1 probe 4T1 cells were seeded in 6-well plates and incubated for 24 h. The cells were then treated with PBS, AC, AC with 660 nm, ACE with/without 660 nm, ACEF with/without 660 nm for 48 h. The medium was then replaced by serum-free medium containing 10 µM DCFH-DA or 10 μg/mL JC-1 for 20 min. The cells were collected and then washed 3 times with serum-free medium. Flow cytometer and inverted fluorescence microscope were used to measure the fluorescence intensity.

## **15. Animals and tumor models**

BALB/c mice and BALB/c nude mice (4-6 weeks) were used to conduct all the *in vivo* studies. The animal experiments were approved by the Animal Ethics Committee of Xi'an Jiaotong University (XJTUAE2024-2371). The mice were housed at Laboratory Animal Center of Xi’an Jiaotong University in a specific pathogen-free atmosphere. All the mice studies were performed according to regional authority guidelines. For the xenograft model, the mice were subcutaneously inoculated at right flank with 200 µL of 4T1 cell suspension (2 × 10^7^ cells/mL). Tumor volume was measured daily as (A × B^2^)/2, where A is the longer diameter and B is the shorter diameter. Mice were randomly grouped into seven groups. Body weight and tumor volume of each mouse were registered daily. Following 15 days of continuous treatment, the mice were euthanized. The tumors and tissues were collected and weighed. For the metastasis model, each mouse was injected via the tail veins with 200 µL 4T1 cell suspension (1 × 10^7^ cells/mL). After 7 days, mice were randomly divided into seven groups (three mice in each group). After 21 days of continuous treatment, the mice were sacrificed. The lung, heart, liver, spleen and kidney were collected and weighed. Metastatic lung nodules were counted and further confirmed via hematoxylin and eosin (H&E) staining.

## **16. H&E staining and immunohistochemistry assay**

The lung, heart, liver, spleen and kidney tissues were fixed in 4% paraformaldehyde and embedded in paraffin, and sectioned at 5 µm thickness. Sections were deparaffinized in xylene, rehydrated through graded ethanol, and stained with hematoxylin for 10 min. After differentiation with acid alcohol and bluing with ammonia water each for 30 seconds, sections were counterstained with eosin for 3 min. Slides were dehydrated, cleared with xylene, and mounted with neutral balsam.

For IHC, paraffin sections of lung tissues were deparaffinized and rehydrated as above, subjected to antigen retrieval in citrate buffer, and treated with 3% H₂O₂ for 10 min to block endogenous peroxidase activity. Samples were incubated with primary antibodies at 4 °C overnight, followed by HRP-conjugated secondary antibodies. After visualization using diaminobenzidine (DAB), nuclei were counterstained with hematoxylin and images were acquired under a microscope.

## **17. Statistical analysis**

The values were expressed as mean ± standard deviation (SD) from three or more independent experiments. One-way analysis of variance (ANOVA) was used to analyze statistical differences between multiple groups, followed by appropriate post hoc tests. An unpaired Student’s *t*-test was used for comparisons between two groups. *p* < 0.05 was considered statistically significant.

**Fig. S1.** The ^1^H NMR spectrum of MEA-EGTA-PEG-FA.

**Fig. S2.** HRTEM and FFT analysis of AC NPs.

**Fig. S3**. X-ray diffraction (XRD) patterns of Au NPs and AC NPs.

**Fig. S4.** EPR spectrum of AC showing oxygen-vacancy/defect-related electronic states.

**Fig. S5**. The thermogravimetric analysis (TGA) of AC and ACEF

**Fig. S6.** Fluorescence spectra of FITC-BSA and ACEF after co-incubation with FITC-BSA: (a) FITC-BSA; (b) ACEF.

**Fig. S7.** Photothermal conversion efficiency (PCE) of different materials under 660 nm laser irradiation. Error bars represent mean ± SD (n = 3).

**Fig. S8**. ROS generation of ACEF under acidic (pH 6.8) and neutral (pH 7.4) conditions using TMB as ROS indicator.

**Fig. S9**. ROS generation measured by TMB-based colorimetric assay: (a) Aggregated free Au+CeO_2_ physical mixture and aggregated AC hybrid nanoparticles under standard conditions without NIR irradiation; (b) Aggregated free Au+CeO_2_ physical mixture with and without NIR irradiation; (c) Aggregated AC hybrid nanoparticles with and without NIR irradiation.

**Fig. S10**. Time-dependent changes in catalytic state and ROS generation of ACEF: (a) Ce^3+^/Ce^4+^ ratios of ACEF after co-incubation with H_2_O_2_ for 30 min, 1 h, and 3 h, followed by NIR photothermal treatment for 5, 10, and 30 min, as determined by XPS; (b)·OH generation efficiency of ACEF under the corresponding conditions, measured by TMB colorimetric assay after NIR irradiation.

**Fig. S11.** Ce 3d XPS analysis of H_2_O_2_-treated ACEF aggregates after incubation under tumor-relevant GSH-containing acidic condition (pH 6.8) for 30 min.

**Fig. S12.** The pH value of different treatment groups changed with time.

**Fig. S13.** ROS generation of ACEF under biologically relevant competitive conditions, measured by UV–vis absorbance at 652 nm using TMB as the indicator: (a) different concentrations of GSH with and without NIR irradiation; (b) different dissolved oxygen conditions, including normoxia (7 mg/L), hypoxia (3 mg/L), and near-anoxia (0.5 mg/L), with and without NIR irradiation; (c) the combined condition of high GSH (10 mM) and low dissolved oxygen (0.5 mg/L), with and without NIR irradiation. Error bars represent mean ± SD (n = 3).

**Fig. S14.** (a) UV–vis spectra of mixed solutions of Ce6 and DPBF after NIR irradiation for different durations. (b) UV–vis spectra of mixed solutions of Ce6, ACEF, and DPBF after NIR irradiation for different durations.

**Fig. S15.** UV–vis spectra of ACEF NPs after incubation in PBS for 0 and 72 h.

**Fig. S16.** Dynamic light scattering (DLS) profiles (a), Zeta potentials (b), and Transmission electron microscopy (TEM) images (c) of ACEF NPs after incubation in PBS containing 5% FBS for 0, 24, 48, and 72 h.

**Fig. S17.** Calcium capture of ACEF incubated in PBS for 72 h containing Ca^2+^, esterase with Ca^2+^. Error bars represent mean ± SD (n = 3).

**Fig. S18.** UV–vis spectrometry of ACEF incubated in PBS for 72 h containing Ca^2+^, esterase with Ca^2+^.

**Fig. S19.** Photothermal curves of ACEF incubated in PBS for 72 h containing Ca^2+^, esterase with Ca^2+^.

**Fig. S20.** Specific calcium capture of ACEF incubated in PBS for 72 h containing major divalent ions (Ca^2+^, Fe^2+^, Mg^2+^). Error bars represent mean ± SD (n = 3).

**Fig. S21.** UV–vis spectra of ACEF after incubation in PBS for 72 h in the presence of Ca^2+^ and esterase under the indicated conditions (TMB, H_2_O_2_, or NIR irradiation).

**Fig. S22.** The ·OH scavenged ratio of ACEF incubated in PBS for 72 h. Error bars represent mean ± SD (n = 3).

**Fig. S23.** The O_2_^-^· clearance ratio of ACEF incubated in PBS for 72 h. Error bars represent mean ± SD (n = 3).

**Fig. S24.** 7day evaluation of ACEF against nonspecific aggregation under different non-triggering conditions. (a) Dynamic light scattering (DLS), (b) UV-vis spectra, and (c) zeta-potential analysis of ACEF NPs after incubation in PBS buffer (pH 6.8), PBS buffer (pH 7.4), 5% FBS-containing PBS, and 300 mM NaCl solution for 7 days. Freshly prepared ACEF NPs were used as the control.

**Fig. S25.** Quantitative statistics of cellular uptake fluorescence. The mean fluorescence intensity (MFI) was measured using Fiji software. Data are presented as mean ± SD (n = 3). Statistical analysis was performed using one-way ANOVA, followed by Tukey's post hoc test for multiple comparisons. ****P* < 0.001, **P* < 0.05.

**Fig. S26.** CLSM images of 4T1 cells co-stained with ACEF-FITC (green), LysoTracker Red (red), MitoTracker Red (red) and Hoechst (blue) at 45 min and 2 h post-incubation. (a) Representative image. Scale bar = 5 µm. (b). Quantitative analysis of the colocalization between ACEF and Lysosomes or Mitochondria. The Pearson’s correlation coefficient (Pearson's R value) was calculated from the indicated regions of interest using the Coloc 2 plugin in Fiji software. Data are presented as mean±SD, (n = 3). Statistical significance was analyzed by unpaired Student’s t-test. ****P* < 0.001, **P* < 0.05,

**Fig. S27.** Lysosomal escape of ACEF and time-dependent intracellular Ca^2+^ regulation in 4T1 cells. (a) Representative CLSM images of 4T1 cells after incubation with ACEF-FITC for different times. Cells were co-stained with ACEF-FITC (green), LysoTracker Red (red), and Hoechst (blue). Scale bar = 5 µm. (b) Flow cytometry analysis of intracellular Ca^2+^ levels in 4T1 cells after treatment with ACEF for 0 h, 0.5 h, 1 h, 2 h, 4 h, 6 h and 8 h, using Fluo-4 AM staining. (c) Quantitative comparison of lysosomal escape efficiency and intracellular Ca^2+^-associated Fluo-4 fluorescence over time. The lysosomal escape efficiency was calculated as (1 − M) × 100%, where M represents the Manders’ overlap coefficient of ACEF-FITC with lysosomes. Data are presented as mean ± SD (n = 3).

**Fig. S28**. Aggregation-associated activation of ACEF in 4T1 cell lysate: (a) UV–vis spectra of ACEF NPs after incubation in 4T1 cell lysate; (b) DLS analysis of ACEF NPs after incubation in 4T1 cell lysate; (c) photothermal heating curves of ACEF after incubation in 4T1 cell lysate.

**Fig. S29.** Flow cytometry analysis of CD86 expression in macrophages under different microenvironmental conditions after ACEF-NIR treatment. (a) CD86 staining of RAW264.7 macrophages cultured with conditioned medium collected from treated 4T1 cells under acidic (pH 6.8) and neutral (pH 7.4) conditions. (b) CD86 staining of THP-1-derived macrophages stimulated with LPS + IFN-γ under acidic (pH 6.8) and neutral (pH 7.4) conditions.

**Fig. S30**. Relative cell viability of 4T1 cells after various treatments. Data are presented as mean ± SD (n=4). ****P* < 0.001, **P* < 0.05.

**Fig. S31.** *In vivo* dynamic biodistribution and quantitative analysis of DiR-labeled ACE and ACEF NPs. Data are presented as mean±SD, (n = 3). Statistical significance was analyzed by unpaired Student’s t-test. ****P* < 0.001, ^###^*P* < 0.001, **P* < 0.05.

**Fig. S32.** (a) *In vivo* infrared thermal images of tumor-bearing mice after intravenous injection of ACF-AIE and ACEF-AIE at 0, 6, 12, and 24 h post-injection, captured after NIR irradiation. (b) *Ex vivo* fluorescence images of tumor tissues harvested at the corresponding time points in (a), showing AIE fluorescence intensity to reflect nanoparticle aggregation status. The scale bar is 50 µm. (c) Quantitative analysis of tumor temperature elevation and AIE fluorescence intensity of ACEF-AIE at different time points. Data are presented as mean ± SD (n = 3). Statistical significance compared to the baseline (0 h) was determined using one-way ANOVA followed by Dunnett’s post-hoc test. ****P* < 0.001, ***P* < 0.01, ns: not significant.

**Fig. S33.** Quantitative analysis of tumor mass. Data are presented as mean ± SD (n = 5). Statistical significance compared to the PBS control group was determined using one-way ANOVA followed by Dunnett’s post-hoc test. ****P* < 0.001, ***P* < 0.01, **P* < 0.05.

**Fig. S34.** Quantitative statistics of ROS levels in tumor tissues. Data are presented as mean ± SD (n = 3). Statistical significance compared to the PBS control group was determined using one-way ANOVA followed by Dunnett’s post-hoc test. ****P* < 0.001, ***P* < 0.01, **P* < 0.05,

**Fig. S35.** Quantitative statistics of ROS levels in normal tissues. Data are presented as mean ± SD (n = 3). Statistical significance compared to the PBS control group was determined using one-way ANOVA followed by Dunnett’s post-hoc test. ****P* < 0.001.

**Fig. S36**. Flow cytometry analysis of immune cell infiltration in tumor tissues. Representative flow cytometry plots and corresponding quantitative analysis of (a) M2 macrophages, (b) M1 macrophages, (c) mature dendritic cells, and (d) CD8^+^ T cells in tumors. Data are presented as mean ± SD (n = 5). ***P* < 0.01, **P* < 0.05.

**Fig. S37**. Flow cytometry analysis of immune cell infiltration in adjacent normal tissues. M1 macrophages (a), mature dendritic cells (b), CD8^+^ T cells (c), and M2 macrophages (d) in adjacent normal tissues. Data are presented as mean ± SD (n = 5). **P* < 0.05.

**Fig. S38.** In vivo metabolic behavior and biodistribution of ACEF. (a) Normalized Au concentration in blood after intravenous injection of ACEF. (b) Biodistribution of ACEF in major organs (heart, liver, spleen, lung, kidney) of mice at 24 h post-injection, determined by Au content via ICP-MS. (c, d) Cumulative Au excretion in feces (c) and urine (d) at different time points post-injection, reflecting hepatobiliary and renal excretion pathways, respectively. Data are presented as mean ± SD (n = 3).

**Fig. S39**. Effects of fresh mouse serum on ACEF: (a) UV–vis spectra of ACEF before and after incubation with fresh mouse serum; (b) photothermal heating curves of ACEF before and after incubation with fresh mouse serum.

**Fig. S40.** Effects of tissue and cell lysates on ACEF: (a) UV–vis spectra of ACEF before and after incubation with liver, spleen, lung, kidney, and 4T1 cell lysates; (b) photothermal heating curves of ACEF before and after incubation with liver, spleen, lung, kidney, and 4T1 cell lysates.

**Fig. S41.** Routine hematology parameters of mice before and after ACEF injection. The white blood cell count (WBC), lymphocyte count (Lymph#), granulocyte count (Gran#), red blood cell count (RBC), platelet count (PLT), and hemoglobin (HGB) levels were measured in mouse blood samples collected before injection (0 d) and at 1 d, 7 d, 14 d post-ACEF injection. Data are presented as mean ± SD (n = 5).

**Fig. S42**. Effects of ACEF on serum Ca^2+^ of mice. Data are presented as mean ± SD (n = 5).

**Fig. S43**. Effects of ACEF on coagulation function of mice. Data are presented as mean ± SD (n = 5).

**Fig. S44**. Effects of ACEF on representative serum metal ion levels of mice. Data are presented as mean ± SD (n = 5).

**Fig. S45.** Liver and kidney function indicators of mice before and after ACEF injection. Serum levels of alanine aminotransferase (ALT), aspartate aminotransferase (AST), creatinine (CREA), total bilirubin (TBIL), and urea (UREA) were measured in mice before injection (0 d) and at 1 d, 7 d, 14 d post-ACEF injection. Data are presented as mean ± SD (n = 5).

**Fig. S46.** Serum levels of tumor necrosis factor-α (TNF-α), interleukin-6 (IL-6), and interferon-γ (IFN-γ) in mice before and after ACEF injection. Samples were collected before injection (0 d) and at 1, 7, and 14 d post-injection. Data are presented as mean ± SD (n = 5).

**Fig. S47.** Immunohistochemical staining of inflammatory cell markers in major organs of mice after ACEF injection. Heart, liver, spleen, lung, and kidney tissues were collected at Days 0 and 14 post-ACEF injection, and stained with CD45^+^ (total leukocytes), Ly6G^+^ (neutrophils), F4/80^+^ (macrophages), and CD3^+^ (T cells). The scale bar is 50 μm.

**Fig. S48.** Organ coefficients (organ weight/body weight) of heart, liver, spleen, lung, kidney and brain were calculated using tissues collected before injection (0 d) and at 1 d, 7 d, 14 d post-ACEF injection. Data are presented as mean ± SD (n = 5).

**Fig. S49.** H&E staining of major organs in mice before and after ACEF injection. Heart, liver, spleen, lung, kidney and brain tissues were collected before injection (0 d) and at 1, 7, 14 days post-ACEF injection, and subsequently subjected to H&E staining. The scale bar is 100 μm.
